# Changes in interstitial fluid flow, mass transport and the bone cell response in microgravity and normogravity

**DOI:** 10.1038/s41413-022-00234-9

**Published:** 2022-11-21

**Authors:** Fei Wei, Kendal Flowerdew, Michael Kinzel, Luigi E. Perotti, Jackson Asiatico, Mahmoud Omer, Candice Hovell, Veerle Reumers, Melanie J. Coathup

**Affiliations:** 1 Biionix Cluster, Department of Internal Medicine, College of Medicine, University of Central Florida, Orlando, FL USA; 2 Marian University College of Osteopathic Medicine, Indianapolis, IN USA; 3 Department of Mechanical and Aerospace Engineering, University of Central Florida, Orlando, FL USA; 4 Imec USA, 194 NeoCity Wy, Orlando, FL USA

**Keywords:** Bone, Bone quality and biomechanics

## Abstract

In recent years, our scientific interest in spaceflight has grown exponentially and resulted in a thriving area of research, with hundreds of astronauts spending months of their time in space. A recent shift toward pursuing territories farther afield, aiming at near-Earth asteroids, the Moon, and Mars combined with the anticipated availability of commercial flights to space in the near future, warrants continued understanding of the human physiological processes and response mechanisms when in this extreme environment. Acute skeletal loss, more severe than any bone loss seen on Earth, has significant implications for deep space exploration, and it remains elusive as to why there is such a magnitude of difference between bone loss on Earth and loss in microgravity. The removal of gravity eliminates a critical primary mechano-stimulus, and when combined with exposure to both galactic and solar cosmic radiation, healthy human tissue function can be negatively affected. An additional effect found in microgravity, and one with limited insight, involves changes in dynamic fluid flow. Fluids provide the most fundamental way to transport chemical and biochemical elements within our bodies and apply an essential mechano-stimulus to cells. Furthermore, the cell cytoplasm is not a simple liquid, and fluid transport phenomena together with viscoelastic deformation of the cytoskeleton play key roles in cell function. In microgravity, flow behavior changes drastically, and the impact on cells within the porous system of bone and the influence of an expanding level of adiposity are not well understood. This review explores the role of interstitial fluid motion and solute transport in porous bone under two different conditions: normogravity and microgravity.

## Introduction

The negative effects of spaceflight on human health are known to involve multiple biological stressors, including microgravity, radiation, loss of the light-dark cycle and confinement. Among these, microgravity exposure (between 10^−3^-10^-5^ *g*) has been reported to have the strongest impact on human physiology and psychology.^1^ While the history of human space flight has focused primarily on the development of research facilities located in Lower Earth Orbits, such as Skylab, Salyut, Mir, and most recently the International Space Station (ISS), there has been a recent shift toward pursuing territories farther afield, aiming at near-Earth asteroids, the Moon, and Mars. Acute skeletal loss has severe implications for long-term (>5 months) inhabitants of the ISS and will be a hindrance to space exploration, as up to one half of bone mass could be lost during a 3-year trip to Mars, resulting in mission-compromising low-energy bone fractures, complications from renal stones caused by skeleton-released calcium and an increased incidence of fragility fractures when returning to full or partial gravity.^2^ This extreme and accelerated bone loss is 10-fold greater than postmenopausal osteoporosis, and it remains mechanistically elusive as to why there is such a magnitude of difference between bone loss on Earth and loss in microgravity.

When in space, the lack of gravity eliminates a critical primary mechano-stimulus, and when combined with exposure to both galactic and solar cosmic radiation, additive injury to healthy human tissue function ensues.^3,4^ An additional and detrimental effect may involve changes in dynamic fluid flow within tissue. Presently, there is only limited information about how changes in this key cell regulator may orchestrate bone turnover during long-duration space travel. Gravity strongly affects fluid behavior by creating forces that drive and alter its motion. In the presence of gravity, fluid flow can also lead to altered phase interactions and processes that regulate gases. Controlling fluid flow in the absence of gravity creates both significant and novel challenges, where flow can be significantly complicated by temperature, capillary networks of different geometries, changes in fluid surface tension, droplets, and undesirable bubble formation. The near elimination of buoyancy, hydrostatic pressure and sedimentation cause adjustments to flow dynamics: liquids climb container walls, there is limited drainage of liquids, and liquids of different densities can stratify.^5–8^ These are a few examples of the many complications induced by microgravity. Such complex multiphase and interfacial flow processes as well as phase separation and unavoidable alterations in particle biodistribution relate to the performance of biosystems. Currently, the impact of these changes within the porous system of bone, along with subsequent changes encountered at the cellular level, are not well understood. The aim of this review is to explore our current understanding of interstitial fluid motion and solute transport in two different conditions, normogravity and microgravity, and to determine how microgravity may influence the subsequent characteristics and behavior of bone tissue fluid when in space. We examine the hypothesis that microgravity may further deteriorate bone architecture through decreased fluid-induced mechanostimulation, altered mass transport and cytoskeletal changes.

## Normogravity

### Interstitial fluid flow and mass transport within the soft tissue extracellular matrix

Interstitial fluid consists of a water solvent (92%) containing amino acids, sugars, salts, fatty acids, coenzymes, hormones, neurotransmitters, minerals, and cell waste products.^9,10^ It accounts for 20% of the water in the human body and up to 12% of body mass.^11^ Interstitial fluid exists either as fluid bound by physico-chemical forces to extracellular matrix (ECM) components (e.g., heparan sulfate and other glycosaminoglycans) or as free fluid moving through the cellular biological medium. The size distribution of the components within the fluid ranges from small molecules and ions (<1 nm) to 10 nm proteins such as albumin, lipoproteins ∼20 nm in size, and fibrinogen (∼30 nm).^12^ In the body, electrostatic forces and energies (e.g., ion pairs, hydrogen bonds) are essential for the interaction of virtually all biological macromolecules.^13^ Due to the polar nature of water, the intercellular and intracellular interactions between water and hydrophilic and hydrophobic molecules, including polysaccharides, lipids, and proteins, are critical for healthy physiological processes. Furthermore, spatial control, ranging from the local control of protein activity and irreversible aggregation to the uptake of pathogens or the management of wastes, are similarly influenced by electrostatic forces and, as such, are essential to many cellular activities.^14^

A simple example of gravity’s impact on fluid flow is the creation of flows due to density differences (buoyancy-induced convection) as well as due to thermal convection. Gravitationally induced bulk convection is a type of natural convection caused by buoyancy variations that result from material properties other than temperature. With gravity, thermal convection occurs when heated fluids rise to the top along the gravity vector, which are then replaced by cooler fluids. Both bulk and thermal convection establish a fluid current in the body that is considered essential to driving mass transport and rapidly dissipating heat.^15,16^ Within the interstitial space, the primary mass transfer mechanism is considered to be molecular diffusion, which is augmented by bulk convection.^17,18^ The work of Swabb et al.^19^ showed that, in soft tissues, the relative importance of convective versus diffusive mass transfer depends on the size of solute molecules, where larger solutes (i.e., molecular weight >1 000 Da) were transported by convection-dominated mass transfer and smaller molecules were transported by diffusion. Notably, this study also showed that fluid velocity was regulated in part by the level of polysaccharides within the interstitium. In soft tissue tumor environments, osmotic and hydrostatic pressure gradients generated by physiologic processes such as drainage toward lymphatics, inflammation, muscle contraction and loading during ambulation were shown to drive flow via dynamic stress through the ECM.^20,21^ Studies have demonstrated that fluid flow can reach velocities between 0.1 and 4.0 μm·s^−1^ within the ECM of soft tissue.^21–23^ Although slow, fluid flow nevertheless plays an important role in nutrient transport, soft tissue maintenance and remodeling, as well as the establishment and maintenance of the microenvironment, where limitations in the supply of vital nutrients lead either to tissue adaptation or necrosis.^24^

### Interstitial fluid flow within the porous bone system

**Fig. 1:**
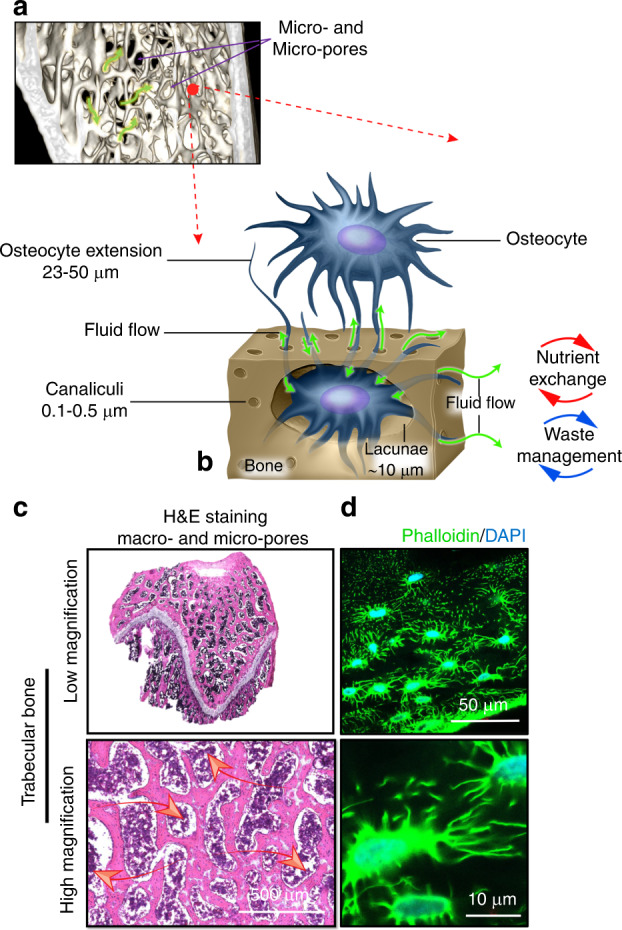
A multilayered porous system in bone allows for dynamic fluid flow (green arrows) at the nano, micro- and macroscale. **a** A microCT image of healthy murine trabecular bone. The bone structure consists of micro- and macropores. **b** A schematic demonstrating osteocytes within the LCS. **c** Representative photomicrographs of a longitudinal section prepared from the distal femur of a healthy rat. The pores are inhabited by bone marrow containing blood vessels and multiple cell groups. The pores provide an environment for fluid movement (red arrows) and the generation of fluid-induced cellular mechanostimulation. **d** Representative images of osteocytes within the LCS. The osteocyte nucleus (blue) and cell processes (green) are observed within the canaliculi and allow communication between cells that are located at distant sites. Images were taken from cryo-sections prepared from healthy rat bone. Not presented are the vascular porosities within the Volkmann and Haversian canals, which provide an additional pore structure, as well as the collagen-hydroxyapatite porosities, which comprise the smallest pore size in bone

Bone is a natural composite material consisting of three primary phases: mineral (mainly hydroxyapatite), organic (~90% Type I collagen) and water. These phases are not interdependent but combine to determine the biomechanical properties of bone. The lacunae-canaliculi system (LCS) within bone tissue and its anatomical parameters vary according to bone type, location, age and health (Fig. 1). The LCS is composed of larger lacunae (~10 µm) and smaller canaliculi (0.1–0.5 µm) inhabited by osteocytes, and this porous system facilitates the exchange of substances, with liquid flow providing nutrients, eliminating metabolic waste and generating fluid shear force to stimulate osteocyte viability and function.^25,26^ Lacunae are roughly tri-axial ellipsoids in mature bone and globular in woven bone.^27^ Canalicular length has been estimated as 23 to 50 µm, with ~41 to 115 canaliculi originating from each lacuna.^28,29^ Spaces between crystallites of the mineral hydroxyapatite and collagen fibers also exist and are estimated to be ~0.01 μm in size.^30^

**Fig. 2:**
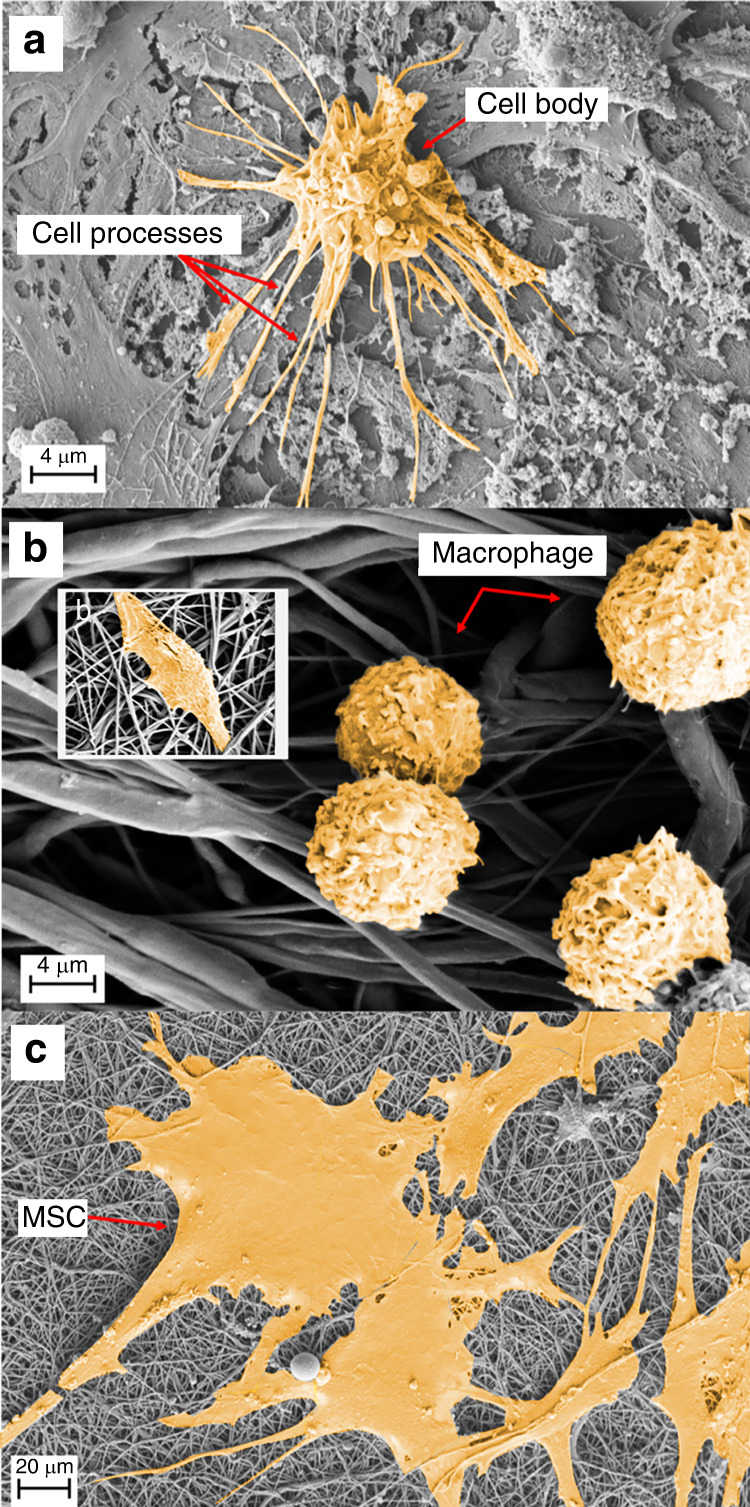
Scanning electron microscopy (SEM) images of cultured cells in vitro. **a** Human osteocytes are stellate in shape and are found within the lacunae-canalicular system. The cell body varies in size between 5 and 20 µm in diameter and sits within the lacunae. Each cell body contains 40–60 cell processes, which are approximately 23–50 µm in length. Cell processes occupy the canaliculi system and establish a communication network. The cell-to-cell distance is approximately 20–30 µm. **b** Image demonstrating rounded M0 murine macrophages, approximately 10-20 µm in size. When activated toward a pro- (M1) or anti-inflammatory (M2) phenotype, cytoplasmic extensions appear, and cell size increases with elongation of the cell body associated with the M2 phenotype (*inset* **b**). **c** Human mesenchymal stem cells (MSCs) are a heterogeneous cell population that are typically large, flat and spindle shaped. MSCs range between 15 and 40 µm in size

In addition to the LCS, cortical bone contains a vascular porosity via the larger-scale Volkmann canals and Haversian systems (~20 μm radius). Trabecular bone consists of a series of micropores ranging between 0.01–20 μm, with macropores 200–500 μm in size. Bone marrow is a cellular soft tissue located within the porous spaces of both cortical and trabecular bone that forms the environment for several cell types. The bone marrow is inhabited by blood vessels as well as multiple cell groups, including osteocytes, osteoblasts, macrophages, adipocytes, endothelial cells, and mesenchymal stem cells (MSCs) (Fig. 2). Oxygen has low solubility in aqueous media and is limited by diffusion distance in most mammalian tissues such that cells are typically located within 100–200 μm from the nearest capillary to ensure efficient gas exchange.^31^

In their pioneering work, Piekarski and Munro in 1977^26^ first theorized that in response to physiological loading, there is fluid flow within the complex interconnected LCS porous network within bone. Until the 1990s, investigators did not identify this network as a mechanosensory organ but instead hypothesized that fluid flow and the subsequent signaling response by cells was due primarily to the receiving of nutrients and removal of waste products. It was not until 1994 that Weinbaum et al.^32^ introduced the hypothesis that bone cells sense mechanical load in response to fluid shear stress, initiating a signal for cellular excitation. It has since been established that the mechanically induced deformation of bone acts as a motive force for fluid displacement, generating fluid pressure gradients that drive interstitial fluid into the LCS and bony macrostructure. The forces generated act directly on bone cells, and this load-induced fluid flow is critical for mechanotransduction as well as enhancing convective solute transport within the macro- and microporosities.^33^ As such, bone tissue would not survive without flow.

Advancements in our understanding of the cellular mechanoresponse to strains, fluid flow velocities and shear stresses are critical. However, these quantities are difficult to measure in situ. There have been many published numerical analyses of fluid flow and mass transport within the LCS as modeled in cortical bone.^34–38^ Verbruggen et al.^39^ employed fluid-structure interaction modeling to develop a complex 3D system that simulated the multiphysics of the mechanical environment of osteocytes in vivo. This study estimated that in a representative state of physiological activity, the average interstitial fluid velocity within the LCS and surrounding the osteocytes was ~60.5 μm·s^−1^, with a maximum shear stress of ~11 Pa. These results are similar to observations reported using experimental tracers in an in vivo mouse model (~60 μm·s^−1^ and ~5 Pa).^40^ However, the bone marrow located within the larger pores that dominate the trabecular structure provides a specialized environment for fluid flow when compared to the liquid flows, pressure distribution, and principles of fluid shear stress within the microscopic LCS. When trabecular bone is subjected to mechanical loading, complexity is introduced through macropore deformation and the interaction between bone and the adjacent soft marrow tissue. Bone marrow is a highly viscous fluid that displays viscoelastic solid properties^41^ and is subject to deformation by the surrounding pores as they change their shape and size under load. These structural changes introduce velocity and pressure gradients that cause cells in the marrow to move relative to one another and to be stretched and deformed, thereby imparting shear forces through intercellular or focal adhesions.^42,43^ Notably, the fat composition and viscosity of bone marrow vary with age and location (40 to 600 mPa•s), and this may also influence the levels of fluid shear stresses generated.^44^ It is therefore conceivable that the subsequent mechanical environment of the marrow within trabecular bone plays a critical role in directing cell activity, function, and fate.^45^ However, there has been little insight into how marrow alters during loading and, subsequently, how the interstitial fluid-induced mechanostimulus to cells is modified. This complex multicellular environment has made the direct study of this microenvironment in situ challenging. Using a finite element model coupled with computational fluid dynamics (CFD), Birmingham et al.^44^ estimated that the load-induced fluid shear stresses in the marrow were between 0.02 Pa and 0.26 Pa under simulated physiological loading. More recently, Metzger and colleagues^46^ used microscale CFD to model fluid velocity within the bone marrow component and demonstrated that during cyclic loading, the volumetric mean marrow velocity averaged ~0.01 mm·s^−1^, applying a shear stress to cells that ranged from 1.67 to 24.55 Pa. Similarly, a numerical bone simulation model developed by Yao et al.^47^ estimated that interstitial fluid flow induced a shear stress with a magnitude up to 30 Pa on the membrane of cells where parameters such as blood pressure, capillary density, capillary permeability, capillary orientation, interstitial pressure and interstitial porosity all affected the applied shear stress and the efficacy of substance exchange.

### The role of the cytoskeleton

The mechanical aspects of cell life are central for cell motility, cell division, intracellular transport, and positioning of organelles, to name a few relevant phenomena.^48^ Therefore, gravity and external mechanical stimuli, including tensile and compressive stresses, fluid-exerted shear, and hydrostatic pressure, all greatly influence the growth, development, and maintenance of healthy tissues and cells.^49^ The cellular response to these forces can be described by 3 sequential events: *mechanosensing* involves the cell sensing changes in its local mechanical environment through changes to the cytoskeletal architecture; *mechanotransduction* involves the conversion of force- or geometry-induced changes into biochemical signals; and the *mechanoresponse* can be short- and/or long-term. Short-term responses include alterations to cell motility systems responsible for migration and surface adherence.^50^ Long-term responses include modifications to the cell leading to altered cell survival or new deposition/remodeling of extracellular matrix.^51^ The overarching term “mechanotransduction” refers to the set of mechanisms that enables the cell to convert a mechanical stimulus into biochemical activity. All osteogenic cells, from MSCs to osteoblasts to osteocytes, are mechanosensitive and therefore can sense and respond to applied force.^52^

**Fig. 3:**
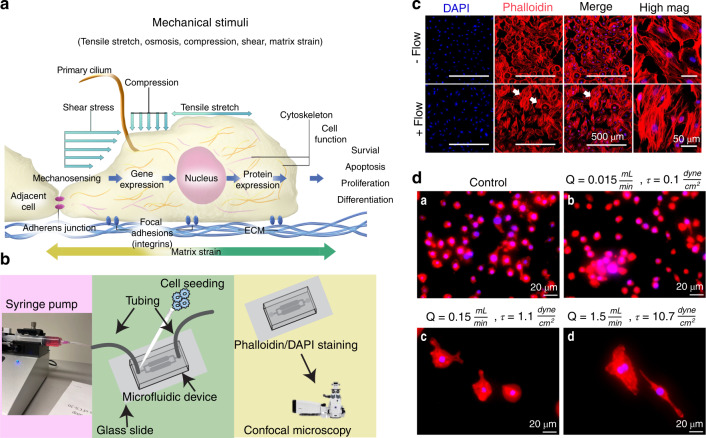
Gravity and external mechanical stimuli influence the growth, development, and maintenance of healthy tissues. **a** Cells sense their mechanical environment (e.g., tensile stretch, compressive strains, and shear stimuli) via mechanisms involving cilia, adherens junctions, ion channels, and focal adhesion. **b** The controlled fluid environment provided by 2D microfluidic devices is a commonly used method to examine the cell response under different flows. **c** Representative micrographs of IDG-SW3 murine late osteoblasts/preosteocytes taken using confocal microscopy showing cell nuclei (DAPI (blue)) and F-actin cytoskeletal filaments [phalloidin (red)]. Cells were cultured within a microfluidic device. A flow rate of 0.15 mL·s^−1^ was applied, and the mechanosensitive actin filaments responded by realigning their structure, becoming more parallel in orientation (white arrows). Images show the fluid-induced response after 24 h of culture. **d** Representative micrographs of macrophages within microfluidic devices (24 h) and examined using fluorescence microscopy (×20 mag.). Images show red cytoplasmic actin filaments (phalloidin) and blue nuclei (DAPI). **a** Static-flow conditions and following the application of continuous fluid flow delivered at **b** 0.1 dyn per cm^2^, **c** 1.1 dyn per cm^2^ and **d** 10.7 dyn per cm^2^ (physiological) fluid shear to cells. The cells were observed to respond differently to changes in fluid shear. M0 (nonactivated) macrophages are characterized by their small size (~10 µm) and abundant number, and this phenotype is indicated in the unstimulated static control group. Following the application of an extremely low fluid shear (0.1 dyn per cm^2^), the cells were fewer in number and slightly larger (~15–20 µm), displaying a more M1-like, proinflammatory, osteodestructive phenotype. Notably, there were fewer nuclei, suggesting cell death. Remarkably, when exposed to higher shear rates, the cells become much larger (~80–100 µm) and are round or spindle shaped, suggesting an osteoprotective M2 phenotype

The cell cytoplasm is not a simple liquid. The cell is a mechanical machine, and continuum mechanics of the fluid cytoplasm and the viscoelastic deforming cytoskeleton play key roles in cell physiology.^48^ Cellular deformations are perceived via complex and intricate regulatory pathways, activating one or more putative mechanosensitive structures, which include adhesion molecules and adhesion complexes (transmembrane integrins, cadherins, and connexins), the cytoskeleton, primary cilia, lipid rafts, stretch-activated ion channels, G protein-coupled receptors, and the nucleus^52^ (Fig. 3). These interactions form crucial links in mechanical continuity that couple the inside of the cell to the outside environment. Virtually all bone cells express the necessary tools, including the primary cilium,^53^ and their interdependent roles have previously been comprehensively reviewed.^52^ In general, fluid shear stress can induce deformation of the bone cell membrane and alteration of membrane proteins, opening mechano-activated ion channels to allow the influx of cations, such as Ca^2+^, Na^+^, and K^+^, into the cell.^52^ Stretch-activated ion channels include the DEC/ENAC family of cation channels (named after *Caenorhabditis elegans* degenerins and mammalian Na^+^ channels), L-type (osteoblasts) and T-type (osteocytes) voltage-sensitive calcium channels, and annexin V voltage-gated calcium channels.^53^ Specifically, increases in osteoblast, osteocyte,^54^ and MSC^55^ membrane tension induce the opening of PIEZO1 channels. Mechanically activated nonselective Ca^2+^-permeable cation channels of the PIEZO family (PIEZO1 and PIEZO2) are recognized as the most important mediators of mechanotransduction and are crucial for bone formation.^56^ Mechanical stimulation enhances calcium flux into the cell, and the resulting calcium spikes mediate osteogenesis.

The cytoskeleton is a mechanosensitive structure and consists of a network of actin, microtubules, and intermediate filaments, which provide shape and stability to cells and connect the ECM to the cell nucleus.^50^ A recent study identified the cytoskeleton as an emerging key player in initiating mechanotransduction.^57^ Actin and microtubule polymers are stiff filaments that constantly assemble and disassemble, with lifetimes on the order of seconds or minutes. The actin cytoskeletal components are comprised of filamentous F-actin, helical G-actin, and actin-binding proteins and act by sensing mechanical force with the subsequent generation of cytoskeletal contractile and protrusive forces. Microtubules are protofilaments containing α and β tubulin heterodimers. Presently, there is no evidence that microtubules function as mechanosensors. However, they do play an indirect role in the mechanoresponse by regulating force-controlled spindle organization, chromosomal alignment, and segregation during mitosis.^50^ Additionally, cells under mechanical stress have been shown to demonstrate microtubule outgrowths along the periphery of the cell. Intermediate filament proteins form both homodimers and heterodimers and are considered the most stable cytoskeletal filaments, serving as sensors of mechanical force direction and strength.^58^ Both actin filaments and intermediate filaments communicate with the external environment via adhesion complexes, which are bound to adhesion receptors. These adhesion complexes sense mechanical forces, relaying information to the cytoskeletal elements within the cell.^58^ When focal adhesion complexes on the cell surface are stimulated by a mechanical signal, such as fluid shear stress, a number of pathways become activated within the cell. Specifically, there is a clustering of transmembrane integrins, integrin recruitment of proteins, and activation of signaling cascades,^58^ thereby communicating stimuli external to the cell nucleus and resulting in responsive changes in gene expression.^58,59^ Significant differences have been reported in terms of the cytoskeletal organization between cell types in bone. For example, MSCs comprise many thick actin bundles, while osteoblasts have fewer filaments and show a thin but dense meshwork of actin.^52^ Fluid shear stress-induced osteogenic differentiation of MSCs is reported to occur via the critical contribution of the actin cytoskeleton.^60^ When osteoblasts were stimulated by fluid shear stress, Pavalko et al.^61^ reported reorganization of actin filaments, and Malone et al.^62^ demonstrated that the response of osteoblasts to fluid shear stress was altered following disruption of the actin cytoskeleton; a similar response has been reported when the microtubule network is broken.^63^ Chen and Jacobs^64^ demonstrated that disruption of the microfilament assembly/disassembly process prevented the fluid flow-induced osteogenic differentiation of MSCs. Additionally, cilia, microtubule-based organelles projecting from the cell surface, have been found to convert fluid flow into a cell response without input from calcium channels or other stretch-activated channels.^65^ In particular, cilia have been linked to osteogenic gene expression and protein secretion in osteocytes, osteoblasts, and mesenchymal stem cells; however, their mechanism of mechanosensation has not been clarified in bone.^66^

Fluid-induced biochemical events initiate intracellular signaling, and the mitogen-activated protein kinase (MAPK)/focal adhesion (FAK) signaling, Ras homolog gene family member A (RhoA) and its downstream effector Rho-associated protein kinase (ROCK) pathways, and the calcium and beta-catenin signaling pathways are considered to dominate events at the transcriptional level in the nucleus.^52^ Notably, the Hippo-TAP/TAZ signaling pathway (Yes-associated protein (YAP); transcriptional coactivator with PDZ-binding motif (TAZ)) has also recently been characterized as an important sensing pathway in bone.^67,68^ In summary, these signaling pathways activate genetic programs, enabling cells to mount evolved responses to mechanical stimuli, thereby establishing cell-regulated feedback loops that maintain tissue homeostasis.^69^

### Mechanical cues and bone cell activity

#### Dynamic loading of bone

**Fig. 4:**
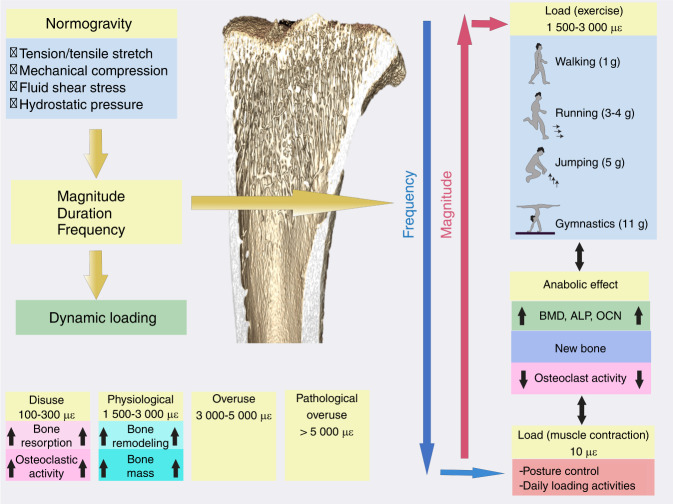
A schematic showing the mechanical loads borne by bone in normogravity. με: microstrain, BMD: bone mineral density, ALP: alkaline phosphatase, OCN: osteocalcin. Several studies have suggested that the rate (determined by the frequency and amplitude) rather than the magnitude alone of the applied loading stimulus correlates with bone formation.^75,247^ This implies that bone formation is enhanced by dynamic loading, and therefore, both the magnitude (or amplitude) and the frequency of loading are important parameters. It has been shown that low magnitude [<10 με (<1 g; 1 g = 9.8 m·s^−2^)] and high frequency (10–100 Hz) loading stimulate bone growth, inhibiting disuse osteoporosis.^248,249^ Peak dynamic strain magnitudes within the physiological range of 1 500–3 000 microstrain (μɛ) are reported to result in bone modeling and an increase in mass. Strain within the disuse range of 100–300 μɛ activates osteoclastic activity and bone resorption. Strain levels above 3 000 μɛ are considered overuse, and those above 5 000 are considered pathological overload^250^

In brief, the complexity and diversity of in vivo mechanical cues present distinct patterns of shear flow, tensile stretch or mechanical compression with various parametric combinations in magnitude, duration and frequency.^70^ Therefore, the osteogenic response to high- or low-impact activity might be related to the response of bone cells either to a sudden increase (i.e., higher rate) or decrease in fluid shear stress, respectively. The rate of loading appears to be critical in bone formation and maintenance; however, it is not well understood how bone cells respond to the rate of loading or what the actual physiological levels of “high” and “low” shear stress are (Fig. 4).

#### Fluid shear stress (FSS)

Cellular responses to shear flow that mimic physiological blood or load-induced interstitial fluid flow have been extensively investigated. In vitro methods duplicate these flows via (i) steady or laminar flow; (ii) pulsatile flow, which introduces changes in flow frequency; and (iii) oscillatory flow, which introduces changes in flow direction. Other factors that have been examined include magnitude, frequency, and length of application.^64^ When investigating the effect of shear stress on human fetal osteoblast cell monolayers, Jacobs et al.^71^ demonstrated that pulsing flow was a much greater stimulator than oscillating flow. The guidance of stem cells toward osteogenic differentiation in 3D bioreactors is also reported to depend on the flow regimen. Liu et al.^72^ observed that intermittent flow (stress alternating from 4.2 dyn per cm^2^ for 1 h to 0.34 dyn per cm^2^ for 11 h) for 14 days significantly enhanced osteogenic gene expression relative to cells cultured in a continuous flow of 4.2 dyn per cm^2^ or static control. A FSS of 4.2 dyn per cm^2^ was produced by a flow rate of 4 mL·min^−1^ for 1 h before being cultured under a fluid flow rate of 0.3 mL·min^−1^ for 11 h. This study showed that a flow rate of 0.3 mL·min^−1^ was too low to stimulate the cells and that the application of intermittent flow had a significant and positive impact on osteogenic differentiation, with the highest levels of ALP, OCN and collagen I (Col-I) expressed on Day 7. Osteocytes that were subjected to a 5-Hz pulse with a mean shear stress of 0.7 Pa, a pulse amplitude of 0.3 Pa and a peak shear stress rate of 8.4 Pa·s^−1^ for 1 h produced a conditioned medium that inhibited the formation of osteoclasts, and the osteocytes were more responsive to flow than osteoblasts or periosteal fibroblasts via NO-dependent pathways.^73^ Correia et al.^74^ investigated the effect of steady and pulsatile medium perfusion on adipose-derived MSCs. The pulsating flow was applied in 12 h intervals, with the interstitial velocity fluctuating between 400 and 1 200 μm·s^−1^ at a 0.5 Hz frequency for 2 h, followed by 10 h of steady flow. A 0.5 Hz frequency was used to resemble the dynamic force spectra applied to the human hip during slow walking.^75^ The results from this study showed that pulsating fluid ranging between 400 and 1 200 μm·s^−1^ was associated with fluctuating shear stresses (0.045–0.134 dyn per cm^2^) and improved early-stage bone formation in comparison to steady flow at 400 μm·s^−1^. Furthermore, the cell response to pulsatile fluid flow was progressively enhanced with the increase of steady flow during culturing.

The directionality of fluid flow was also shown to be important, with cells experiencing unidirectional flow exhibiting different characteristics from cells experiencing oscillatory fluid flow. Furthermore, the velocity of interstitial fluid flow is considered to play a key role in activating surrounding cells when bone is under stress. Physiological levels of fluid flow apply pressure onto the walls of the narrow channels within bone, creating shear stress and ranging in magnitude between 0.8 and 3 Pa (8–30 dyn per cm^2^).^32,59^ Using a parallel plate flow chamber, Yi et al.^76^ investigated protein release from MSCs following the application of a steady flow producing 3 dyn per cm^2^ of low shear stress for 6 h (Table 1). The results showed that MSCs responded to low shear stress, and 32 specific proteins were identified, of which 10 were upregulated. The effect of FSS on osteoblasts is reported to be detectable at 10 dyn per cm^2^,^77^ which is at the lower end of the physiological range (8–30 dyn per cm^2^).^32,59,78^ Riehl et al.^79^ investigated the effect of physiologically relevant shear stresses at 2, 15 and 25 dyn per cm^2^ on MSCs and found that flow shear stress levels had a significant influence on MSC migration. The total displacements, confinement ratio, motility coefficient, and number of cells migrating with the flow over time showed an increasing trend with increasing shear stress. Grayson et al.^80^ investigated the effect of interstitial flow velocity on cell phenotype and the formation of bone-like tissues in 3D engineered constructs. Flow velocities of 80, 400, 800, 1 200 and 1 800 μm·s^−1^ corresponding to estimated shear stresses ranging between 0.6 and 20 mPa were investigated, and the results demonstrated that velocities from 400 to 800 μm·s^−1^ yielded the best overall osteogenic response to MSCs (based on OCN, OPN, bone sialoprotein (BSP) and Col-I expression). Using mathematical models, they determined that the lowest flow velocity of 80 μm·s^−1^ would provide a sufficient oxygen supply (∼0.205 mol·m^−3^) to maintain cell viability; however, this flow rate did not support osteogenesis. Fluid shear stresses ranging between 0.5 and 2 Pa (5–20 dyn per cm^2^) have been widely reported to beneficially impact osteoblasts in vitro,^81^ such as through increased intracellular calcium production and an increased release of prostaglandins.^82^ Furthermore, a study by Yu et al.^83^ investigated the effect of fluid shear stress on the proliferation of MC3T3-E1 osteoblasts and showed that shear ranging within 1.5 to 52.6 µPa promoted cell proliferation and differentiation with increased levels of runt-related transcription factor 2 (RUNX2), whereas shear above 412 µPa inhibited growth. Studies have also augmented osteogenic activity via microflow within microfluidic chips. Leclerc et al.^84^ studied the osteoblast response under 0, 5, and 35 μL·min^−1^ flow within a 3D microchannel and showed that ALP activity was enhanced 7.5-fold under a flow of 5 μL·min^−1^ when compared with the static control. Jang et al.^85^ designed a drug screening device and observed that a microfluidic flow of 0.2 μL·min^−1^ with a shear stress of 0.07 dyn per cm^2^ combined with a bone morphogenetic protein 2 (BMP-2) cue significantly induced the osteogenic differentiation of MC3T3-E1 cells. FSS stimulation of osteoblasts has also been shown to increase cell adhesion by enhancing the affinity of intracellular integrins to extracellular matrix ligands as well as to biomaterial surfaces.^86,87^

**Table 1:** Studies are presented in increasing order of shear stress and according to cell type

Authors	Shear Flow	Pattern	Cell Type	Outcome
Yi et al.^76^	3 dyn per cm^2^, 6 h (low)	Laminar/steady	hMSCs	Increased expression of Annexin A2 (*P* < 0.001), GAPDH (*P* < 0.001)
Arnsdorf et al.^60^	10 dyn per cm^2^, 1 Hz, 1 h	Oscillatory	Murine MSCs	Upregulation of RUNX2, SOX9, PPARγ [all (*P* < 0.01)]. Osteogenic differentiation via RhoA and ROCKII
Correia et al.^74^	400 μm·s^−1^ (1.64 mL·min^−1^; 4-5 mPa shear stress) to 1 200 μm·s^−1^ (4.92 mL·min^−1^; 12.5-15 mPa shear stress), 0.5 Hz for 2 h, followed by 10 h of steady flow over 5 weeks	Pulsating	MSCs	Optimal for osteogenesis was 2 weeks of steady flow with 3 weeks of pulsatile flow. Increased OPN, BSP, PGE2 (all *P* < 0.05).
Xing et al.^232^	5 dyn per cm^2^	Parallel plate	Rat Osteoblasts	Increased Col-I and proliferation. Decreased ALP, proliferation
Kämmerer et al.^77^	10 dyn per cm^2^, 24 h (shear at the center=1 dyn per cm^2^ with 10 dyn per cm^2^ at the periphery)	Rotating at 200 r·min^−1^	Osteoblasts	Actin filaments re-aligned themselves towards orientation of fluid flow
Malone et al.^62^	12 dyn per cm^2^, 1 Hz, 1 h	Oscillatory	Murine Osteoblasts	PGE2 increased 3-fold (*P* < 0.05). No increase in F-actin development
Tan et al.^73^	0.4-1.0 dyn per cm^2^, 5 Hz, 1 h Mean stress of 0.7 Pa	Pulsating 0.3 Pa	Chicken Osteocytes	NO production decreased (*P* < 0.05). Osteoclast formation inhibited via soluble factors in the conditioned medium after 60 mins of exposure
Li et al.^233^	5-50 dyn per cm^2^, 0.5-2 Hz, 1, 2 and 4 h. Peak stress of 0.5, 1, 2 and 5 Pa. oscillating frequency of 0.5, 1 and 2 Hz	Oscillatory	MLO-Y4 Osteocytes	Higher shear stress (5 Pa) and longer flows (4 h) increased COX2 (*P* < 0.05). Increased stress, frequency and duration increased RANKL/OPG ratio.

*GADPH* glyceraldehyde-3-phosphate dehydrogenase, *rpm* revolutions per minute, *NO* nitric oxide, *RUNX2* runt-related transcription factor 2, *ALP* alkaline phosphatase, *PPAR* peroxisome proliferator-activated receptor gamma, *PGE2* prostaglandin e2, *COX2* cyclooxygenase 2, *RANKL* receptor activator of nuclear factor kappa-B ligand, *OPG* osteoprotegerin, *OPN* osteopontin, *BSP* bone sialoprotein

#### Tensile strain

To respond to mechanical stimuli, a cell must first adhere to a surface through focal adhesion junctions. Human bone marrow-derived stem cells respond to active mechanical stimulation, where 2%-8% uniaxial strain through tensile stretching resulted in osteogenic differentiation or subsequent bending resulted in both osteogenic and chondrogenic differentiation^88,89^ (Table 2). Tensile strains between 8% and 12% resulted in reduced proliferation as well as increased expression of RUNX2, ALP, Col-I and BMP-2,^88,90,91^ suggesting the promotion of osteogenic differentiation at these strain levels. Studies have also shown that osteoblasts respond to tensile stretch and that <9% stretch strain promotes human osteoblast proliferation, which is strain-magnitude dependent.^92,93^ However, at lower strain levels (<2.5%), the expression of BMP increased while suppressing the expression of transforming growth factor-β (TGF-β), as well as the activity of ALP and secretion of Col-I,^92^ suggesting a reduction in osteoblast activity. Increased levels of osteoprotegerin (OPG), which is an inhibitor of osteoclastogenesis, have also been reported.^94^ When higher strains of 15% were applied, proliferation increased with decreased expression of ALP and RUNX2, demonstrating an inhibitory effect on osteogenic differentiation and suggesting a higher threshold limit.^95^ Interestingly, MSCs experiencing tension at these higher levels also exhibit reduced expression of adipogenic, chondrogenic, and neurogenic markers such as Col-II, aggrecan, dystrophin related protein 2, and peroxisome proliferator-activated receptor-γ.^96^ When tensile strains between 1 000 and 5 000 μɛ were applied to osteoclasts at a frequency of 0.5 Hz, low-magnitude strain levels (2 000 and 2 500 μɛ) suppressed osteoclastic fusion and activation, while high strains (5 000 μɛ) promoted their fusion and activation.^97^ These results show that varying strain levels have a direct role in regulating both bone-forming and bone-resorbing cells.

**Table 2:** Tensile strain (osteogenic differentiation)

Authors	Tensile Strain	Pattern	Cell Type	Outcome
Huang et al.^90^	3%, 0.1 Hz for 1, 3 or 5 days	Cyclic	hMSCs	Reduced proliferation. Increased FAK phosphorylation. Reorientation of cytoskeleton. Increased cbfa1 (*P* < 0.05), ALP (*P* < 0.05) and mineralized matrix deposition (*P* < 0.05). Fibronectin and laminin coatings enhanced differentiation.
Ward et al.^96^	3%–5%, 0.5 Hz, 2 h·d^−1^, 28 days	Intermittent	hMSCs	Increased BSP2, OCN, Osterix and mineral deposition.
Jagodzinski et al.^88^	2% or 8%, 2 h per 3 times a day, 3 days. 1, 4 and 7 days	Cyclic	hMSCs	2%: increased OCN at 4 days, decreased by Day 7. 8%: increased ALP (*P* < 0.04), OCN (*P* < 0.01) and cbfa1 (*P* < 0.03). Overall effect augmented at 8% stretch.
Haasper et al.^234^	2% or 8%, 1 Hz. 3 times daily for 2 h over 3 days	Cyclic	hMSCs	Increased FosB, RUNX2 (*P* < 0.05). Col-I expression increased with length of treatment (*P* < 0.05). Greater response at 8%.
Shi et al.^235^	3%, 6% or 9%, 0.5 Hz for 1, 3, 7 and 10 days	Uniaxial, cyclic, sinusoidal	hMSCs	Decreased ALP (*P* < 0.05). Largest decrease in 3% group. Decreased RUNX2 and OPN (*P* < 0.05) gene expression but not protein level. Col-I decreased by Day 7.
Zhang et al.^236^	10%, 1 Hz for 96 h	Continuous sinusoidal	Rat BMSCs	Reduced proliferation. Increased RUNX2, ALP, Col I and OCN (all *P* < 0.05).
Sumanasinghe et al.^91^	0, 10% or 12%, 1 Hz, 4 h·d^−1^, 7 or 14 days. Cultured in 3D collagen matrices	Uniaxial, cyclic	hMSCs	BMP-2 expression increased at 10% and 12% at 7 and 14 days (*P* < 0.05). Increased BMP-2 in 12% group at 14 days only (*P* < 0.05).
Koike et al.^95^	0.8%, 5%, 10%, 15%, 24 and 48 h at 1 Hz	Equibiaxial	ST-2 murine MSC cell line	Proliferation increased at 5%, 10% and 15% (*P* < 0.05). ALP and RUNX2 increased at 0.8% and 5% (*P* < 0.05) but decreased at 10% and 15% (*P* < 0.001). Cbfa1/RUNX2 increased at lower magnitudes (0.8% and 5%) and decreased at the higher 15% elongation (*P* < 0.05). OCN decreased at 5%, 10% and 15%. Overall, osteoblastic differentiation increased at low magnitudes.
Wu et al.^237^	0, 3%, 8%, 13% and 18%, 0.5 Hz for 8 h·d^−1^ for 3 days	Cyclic	Murine MSCs	8% strain optimal. Increased ALP and matrix mineralization (*P* < 0.01). Increased BMP-2, RUNX2 (*P* < 0.01).
Qi et al.^89^	2 000 με for 40 mins	Cyclic	Rat MSCs	Increased ALP and cbfa1 (*P* < 0.05)

Studies are presented in increasing order of strain and according to cell type

*hMSCs* human mesenchymal stem cells, *Col-1* collagen-1, *OCN* osteocalcin, *ALP* alkaline phosphatase, *RUNX 2* runt-related transcription Factor 2, *cbfa1* core binding factor alpha 1, *FAK* focal adhesion kinase, *OPN* osteopontin, *cpm* cycles per minute, *TGF-β* transforming growth factor-1, *BGH3* TGF-β1-induced protein ig-h3, *BMP2* bone morphogenetic protein-2

#### Compression

Compression is also a significant stimulus to bone cells. Dumas et al.^98^ applied dynamic compression at various frequencies to MSCs seeded onto hydroxyapatite ceramic scaffolds. The results showed that compression at 3 Hz caused an upregulation of bone-specific proteins. In contrast, frequencies of 50 and 100 Hz reduced osteogenic differentiation and showed the cells to be responsive to both strain and strain rate (Table 3). Jagodzinski and colleagues^99^ applied cyclic mechanical compression with a maximum strain of 10% to seeded MSCs under continuous perfusion and demonstrated an increase in the expression of RUNX2 and OCN, suggesting that the addition of perfusion to compression promoted osteogenic lineage commitment. Hydrostatic pressure can also encourage osteogenic differentiation. Both static (23 kPa) and dynamic hydrostatic pressures (10 to 36 kPa, 0.25 Hz) were capable of inducing osteogenesis in rat bone marrow-derived MSCs.^100^ Using CFD modeling, Anderson et al.^101^ demonstrated that while the osteocyte cell body within the lacunae is exposed to the effects of hydrodynamic pressure, the cell processes within the canaliculi are exposed primarily to shear stress. The shear stress to the processes increases with increasing distance from the cell body.

**Table 3:** Hydrostatic compression

Authors	Mechanical Compression	Pattern	Cell Type	Outcome
Stavenschi et al.^238^	10, 100, 300 KPa, 0.5, 1, 2 Hz, 2 h·d^−1^, 4 days	Cyclic	hMSCs	Increased osteogenic response (COX2, RUNX2, OPN) was magnitude- and frequency-dependent. Optimal was 300 KPa at 2 Hz (COX2 and OPN *P* < 0.05). Low 10 KPa increased RUNX2 at 0.5 Hz over 4 h (*P* < 0.05).
Ravichandran et al.^239^	0.22%, 1 Hz, 4 h·d^−1^ for 4 weeks	Cyclic	hMSCs	Increased osteonectin and Col 1 on Day 7. 3.76-fold increase in ALP on Day 14 (*P* < 0.001). Increased matrix deposition on Day 14.
Jagodzinski et al.^99^	10%, 0.5 Hz, 24 h, 1,2 and 3 weeks	Cyclic w/continuous perfusion	MSCs	Proliferation increased at 2 and 3 weeks in all groups (except control). OCN and RUNX2 increased in all except groups at 1, 2 and 3 weeks (*P* < 0.05).
Chen et al.^240^	0.33, 0.5 and 1 MPa for 4, 6 and 8 h at 1 Hz	Cyclic	Murine MC3T3-E1	Optimal compression was 0.5 MPa for 6 h. No effect seen at 1 MPa. Increased ALP, RUNX2, OCN and osterix (*P* < 0.05). Reduced ALP at 1 MPa (*P* < 0.01).
Wang et al.^241^	30 KPa, 1 Hz for 2, 4 and 8 h	Cyclic	Murine MC3T3-E1	Upregulation of RUNX2, BMP2 and OPN over all timepoints (*P* < 0.01).
Nagatomi et al.^242^	10–40 KPa, 1 Hz, 1 h·d^−1^, 19 days	Cyclic	Osteoblasts	Increased Col 1 and Ca^2+^ (*P* < 0.05).
Priam et al.^243^	1.67 MPa, 1 Hz, 24 h	Cyclic	Murine Osteoblasts/osteocytes	Increased MMP3, MMP13 (*P* < 0.001).
Liu et al.^244^	68 KPa, 0.5 Hz, 1 and 2 h	Cyclic	MLO-Y4 osteocytes	Increased intracellular Ca^2+^ (*P* = 0.002). 4.4-fold increase in microtubule bucking points (*P* = 0.049). Increased COX2 at 1 h (*P* = 0.006). Increased OPG at 1 h.
Sittichockechaiwut et al.^245^	5%, 1 Hz, 2 h·d^−1^, on Days 5, 10 and 15	Cyclic	MLO-A5 osteocytes	Increased cell viability, 2-fold increase in collagen in all loaded groups on Days 10 and 15 (*P* < 0.05). 4-fold increase in calcium in loaded groups by Day 20 (*P* < 0.05). Col-I, OCN and OPN increased by Day 5 (*P* < 0.05).
Kikuta et al.^246^	4.0 g·cm^−2^ (0.4 kPa) 1, 3, 6, 9, 12 & 24 h	Static, uniform compression	Human osteoclasts	Jagged1 (notch signaling), RANKL, TNFa, sRANKL, IL-6, and TRAP all increased (*P* < 0.05).

Studies are presented in increasing order of pressure and according to cell type

*MMP* matrix metalloprotein, *COX2* cyclooxygenase 2, *RUNX 2* runt-related transcription factor 2, *Col-I* collagen I, *OCN* osteocalcin, *OPN* osteopontin, *ALP* alkaline phosphatase, *BMP2* bone morphogenetic protein-2

## Microgravity

### Alterations in fluid motion

Spacecraft such as the ISS orbit at an altitude of approximately 400 km (250 miles) above the Earth’s surface. At this altitude, gravity is 90% as strong as on Earth’s surface. The spacecraft is in a constant state of freefall, resulting in apparent weightlessness. The difference in gravitational pull on the Earth’s surface and in space has a dramatic effect on surface tension and fluid dynamics.^102^ Surface tension is the attraction of molecules within a fluid toward each other, and this attraction competes with gravity on the Earth’s surface.^103^ Surface tension serves as a cohesive force drawing fluid together, while gravity compels it to dip in the center, eventually pulling the molecules apart. On Earth, gravity distorts the shape when a liquid is resting on or attached to a surface. Under the reduced gravity conditions experienced in space, both hydrodynamic shear and hydrostatic pressure are significantly reduced, and surface tension becomes the dominant force; as such, the molecules stay in tight spheres and films, maximizing intermolecular attraction. Furthermore, surface tension causes droplets of any liquid to form almost perfect spheres in the absence of gravity, and the movement of these fluid spheres and films is much slower when compared with movement on Earth.^104^

### Mass transport and protein aggregation

The reduction in gravitationally induced fluid bioconvection may become critical in microgravity. As described earlier, convective fluid flow exists to dissipate metabolic products via mass transport, in particular the larger molecular weight solutes. As such, a reduced rate of fluid convection and a slower solute diffusion process in microgravity may contribute to impaired heat and biomolecule exchange, conceivably facilitating the preferential transport of smaller solutes. Although the pulsing blood pressure within the larger vessels in animal tissues and the muscle contractions that actively deform bone may serve to drive convective flow, a reduced level of fluid convection could still be an anoxic factor to cells localized within tissue where fluid flow is reduced due to microgravity. Few studies have investigated mass transport in normogravity versus microgravity. Liu et al.^37^ developed a multiscale 3D fluid-solid coupled finite element model and estimated a 2-3 order of magnitude reduction in fluid flow and therefore solute transport within the LCS in microgravity. Furthermore, the transport of the simulated particle load differed based on the load frequency, with solute transport increasing as the frequency increased. Using a similar computational model, Wang et al.^38^ reported deficient mass transfer within the LCS in a microgravity environment, especially to osteocytes located at a distance from Haversian canals. The authors concluded that reduced mass transport may contribute to microgravity-induced osteoporosis.

In microgravity, particle sedimentation within fluids is significantly limited, and patterns due to changes in density or the tendency of particles to aggregate become more complex and difficult to predict. Mass transport plays a key role in crystal growth, and many in vitro protein crystallization experiments, where crystals of biological molecules are grown from supersaturated solutions, have been conducted to investigate the influence of microgravity. Certain proteins self-assemble into ordered supramolecular structures, such as crystals and filaments, under specific physiological and pathological conditions.^105^ The aggregation process is influenced by two gravity-driven phenomena: sedimentation of the crystals and natural convection in the feeding solution. Among the proteins investigated were the enzyme lysozyme, the protein canavalin and the transport protein serum albumin.^106^ From these early experiments, it was concluded that crystals grown in space were of a higher quality and generally of greater size than ground-based controls. More recently, Martirosyan et al.^107^ demonstrated that when crystals containing different protein aggregate ratios were grown on the ISS, growth occurred principally by diffusional mass transport when compared with ground-based studies. The results showed the generation of additional nucleation events and the formation of altered crystal dimensions and different mean growth rates. It was speculated that the changes seen were due to lower transport rates for larger aggregates in this convection-limited environment. Furthermore, Bell et al.^108^ investigated molecular self-assembly on the ISS and reported that lysozyme protein fibrils showed a distinctly different morphology. The fibrils formed in microgravity were shorter, straighter, and thicker than those formed in ground-based studies. A recent study by Matsushita et al.^109^ demonstrated that microgravity suppressed amyloid fibril formation by reducing the protein‒protein interaction via decreased fluid convection. The authors concluded that the cytotoxicity of amyloid fibrils may be reduced and that patients with amyloidosis, a protein metabolism disorder, may be more suitable than healthy people to live in space. These results were supported by Yagi-Utsumi et al.,^105^ who reported significant differences in amyloid formation kinetics and fibril morphology between microgravity-grown and ground-grown Aβ(1-40) amyloids. Protein crystallization and fibrilization occurred much more slowly in microgravity, and the authors speculated that the absence of fluid convection combined with a slower diffusion rate resulted in a decreased fibril growth rate. The gravitational influence on molecular aggregation is still not fully understood, and it is unclear whether weak protein interactions are overcome in microgravity, leading to changes in aggregate concentration or in phase separation.

Biomolecules are unique in their properties, both in terms of size and complexity, and give rise to crystals and filaments that also have unique functions.^110^ The in vitro studies described here highlight that mass transport, the mechanism of biologically relevant macromolecular protein crystal formation, and the self-assembly of fibrils in vitro are altered in microgravity compared with normogravity. However, it remains unclear what role, if any, altered biological macromolecule transport and aggregation may contribute to the tissue dysfunction seen in astronauts in vivo. Interestingly, without the gravity-induced loading vector as a guide, Tauber et al.^111^ reported metabolic alterations in primary human macrophages following long-term exposure to microgravity. The results demonstrated that the transcription, translation and organization of cytoskeletal proteins were altered. Modification of their assembly and formation has also been reported by Tabony and Job.^112,113^ Reduced transcription and translation of cytoskeletal and cytoskeletal-associated proteins in osteoblasts has also been reported.^114,115^ Thus, it is plausible that microgravity-induced alterations in fluid flow may promote homeostatic dysfunction and contribute to underlying diseases initiated in space, including expedited bone loss.

### Cytoskeletal changes

Osteoblast cell morphology is significantly altered in microgravity, with an increased cell area and volume and rounder shape reported.^116,117^ Previous studies have theorized that the change in cell size is due to decreased mechanical stiffness and changes in tension within the actin filaments, which promotes cell expansion.^118,119^ Nabavi et al.^118^ reported enlarged nuclei when investigating the influence of microgravity on the nuclei of murine osteoblasts. An increased nuclear size is characteristic of programmed cell death, suggesting that microgravity conditions may promote osteoblast apoptosis.

**Fig. 5:**
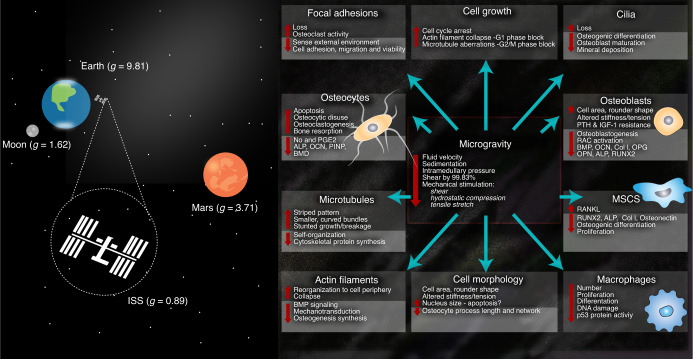
The effect of microgravity on fluid flow within bone and the subsequent cell response reported in the literature. Bone tissue is a complex mechanical environment that provides a specialized habitat for numerous mechanosensitive cell types

In normogravity, microtubules self-organize in a pattern of periodic growth in the direction of gravity.^112,120^ The influence of microgravity on microtubule structure was investigated by Nabavi et al..^118^ Murine osteoblasts were exposed to 5 days of microgravity, and the results demonstrated that the bundles formed were significantly shorter and curved compared to the morphologies seen in normogravity (Fig. 5). Similarly, Tabony et al.^120^ observed that the microtubules became smaller, formed wave patterns and did not have the same ability to self-organize when compared with cells cultured in gravitational conditions. The authors suggested that the lack of gravity either stunted microtubule growth or created an environment that enhanced microtubule breakage. Hughes-Fulford and Lewis^121^ showed that the number of filaments in the cytoskeleton was reduced following exposure to microgravity, and studies have described the formation of striped patterns.^112,122^ Chen et al.^123^ described a similar phenomenon in the formation of actin filaments under microgravity conditions. In the study, the actin cytoskeleton of rodent bone marrow-derived MSCs was reorganized and redistributed into abnormal patterns under both real and simulated microgravity conditions. After 4 days of microgravity exposure, the actin cytoskeleton of osteoblasts had collapsed, significantly impacting multiple downstream signaling pathways, most notably, inhibiting the BMP signaling axis.^115,124^ Due to their collapse, the actin filaments were unable to regulate this process, impairing the mechanotransduction of external signals and limiting osteogenesis. Di et al.^125^ investigated actin filament formation within murine osteocytes and described their reorganization and distribution toward the cell periphery under microgravity. Other studies have reported that exposure to microgravity decreased the expression of actin and actin-associated proteins, namely, Arp2/3 and RhoA, subsequently resulting in the disorganization of the actin cytoskeleton.^126,127^

A prominent effect of gravity-induced hydrostatic pressure is the adhesive compression of the cell against a rigid substrate or to other cells, where the greater the gravitational force is, the more focal adhesions form.^128^ Studies have demonstrated loss of focal adhesions along the osteoblast surface after just 4 days in microgravity.^129,130^ This loss may serve to hinder the cells’ ability to sense changes in the external cell environment, consequently effecting cellular adherence, their migration capacity and viability as well as their response to fluid shear stress and any subsequent bone-forming^124^ or bone-resorbing activity.^118^

One key cellular process that relies heavily on the cytoskeleton is the cell cycle. Both spaceflight and ground-based simulation studies support the theory of cell cycle arrest following microgravity exposure.^129^ When the cytoskeleton is altered, such as it is in microgravity, cell growth can be blocked at either the G1 phase or G2/M checkpoint. Blocks during the G1 phase occur as a result of actin filament collapse, while G2/M checkpoint blocks are reported to occur as the result of microtubule polymerization complications. Finally, cilia, a key link between fluid flow and the cell response, have also been shown to disappear in osteoblasts under microgravity conditions. The microtubules are reported to be depolymerized within the cilia while in microgravity, which inhibits osteogenic differentiation, maturation, and mineralization of the bone cell.^66^

### Alterations in fluid flow

Although body mass, extracellular fluid volume, and plasma volume are reduced during spaceflight and remain reduced upon landing, the changes in total body water are comparatively small. Microgravity may reduce fluid flow within bone via two mechanisms, cephalad fluid shifts and mechanical unloading, and these changes are proposed as mechanisms that in part cause bone loss in astronauts.

#### Cephalad fluid shifts

Under normal gravity conditions, a fluid pressure gradient exists extending from the head to the feet, with pressure being the greatest at the feet and lowest in the head.^131,132^ When exposed to microgravity, this pressure gradient is lost, and the body experiences a uniform fluid pressure of approximately 30 mmHg. Loss of this physiological pressure gradient results in an increased pressure in the upper body and above the heart and a decreased pressure in capillaries below the heart.^131^ As a result, blood flow is significantly reduced to the lower extremities with an increased flow to the head, chest, and upper extremities, compared to the body in normogravity.^133,134^ The most easily visible manifestation of this is swelling of the face and thinning of the legs within a short time of exposure to microgravity.^131^ The vasculature adapts, and blood vessels in the upper body undergo hypertrophy, while blood vessels in the lower body undergo atrophy.^135^ Additionally, in microgravity, the overall blood volume decreases due to shifts in fluid toward the interstitial spaces.^136^ Parallel to the nonuniform changes observed in fluid flow and pressure, bone loss during spaceflight is also not uniform within the body.^131^ Collaren et al.^137^ demonstrated that femoral and tibial perfusion was reduced within 10 minutes of beginning hindlimb suspension (HLS) in rats. The blood flow to these bones continued to decrease for the remainder of the 28 days of HLS. The cortical and cancellous masses in the femur and tibia both decreased over the 28-day course. These findings were reversed in the skull, mandible, clavicle, and humerus, which all demonstrated increased blood flow within 10 minutes of HLS and increased mass after 28 days. Interestingly, blood flow to these bones did not continue to increase but returned to normal 7 days after stopping HLS.

#### Mechanical unloading and reduced interstitial fluid flow

Few studies have investigated changes to interstitial fluid flow within bone when in microgravity, and it remains unclear what range of fluid dynamics stimulate mechanosensory cells to induce an osteoprotective or osteodestructive response. It is conceivable that the range of shear stress necessary to elicit a protective or destructive cell response will be cell-dependent. Due to their activity, exposure of osteocytes, macrophages, and osteoclasts to disadvantageous stresses may be more destructive at the tissue level than the exposure of MSCs and osteoblasts. However, the effects of such extremely low fluid shear stress on bone cells are not fully understood. Using computational modeling, osteocytes, especially within the deeper layers of the lacunae and away from the Haversian canal, were estimated to undergo apoptosis due to a microgravity-induced reduction in fluid velocity and decreased fluid shear force stimulation, resulting in a reduction in bone mass.^121^ Klein-Nulend et al.^138^ theorized that decreased fluid flow caused by extended periods of unloading may have caused osteocytic disuse throughout the entire bone, leading to the accelerated osteoclastic resorption of bone. Amin^139^ hypothesized that because microgravity causes a decrease in hydrostatic pressure, intramedullary pressures were also reduced, leading to decreased fluid shear forces to osteocytes and ultimately bone loss. Yang et al.^140^ investigated the effect of fluid shear stress on murine osteocyte-like cells, and the results showed that nitric oxide (NO) and prostaglandin E2 (PGE2), normally released following the application of shear stress under normogravity conditions, were inhibited in microgravity. Both NO and PGE2 are essential components in the tissue damage cascade, as they signal to enhance cell differentiation and growth. Additionally, this study also reported that three key bone formation biomarkers, ALP, OCN, and procollagen type I N propeptide (PINP), were suppressed or inhibited due to loss of shear stress under microgravity conditions. L-type calcium channels in osteoblasts are stimulated by fluid shear stress, which activates a number of bone formation signaling pathways. Sun et al.^141^ reported that under microgravity conditions, these L-type calcium channels were inhibited in mouse osteoblast-like cells due to an upregulation in microRNA (miR − 103), impairing new bone formation. Furthermore, Gao et al.^142^ demonstrated that the osteogenic differentiation of MSCs was significantly reduced when the cells were exposed to extremely low fluid shear stress (0.01 dyn per cm^2^) compared to higher stresses. In osteoclasts, Gao et al.^143^ showed that osteoclast precursor cells actively migrated toward regions of low-fluid shear stress, and in a later study, they demonstrated that the ratio of tartrate-resistant acid phosphatase-positive mature multinucleated osteoclasts was significantly higher under extremely low-fluid shear stress conditions.^144^

Although not in the context of bone, it has also been shown that 30 minutes of exposure to a low fluid shear stress (0.1 dyn per cm^2^) significantly promoted macrophage polarization toward a proinflammatory phenotype.^145,146^ This may be critical, as macrophages and monocytes are immune cells able to directly regulate bone turnover through the release of either proinflammatory cytokines (e.g., interleukin 1β (IL-1β), interleukin 6 (IL-6), nitric oxide synthase, and tumor necrosis factor-α (TNF-α)), leading to bone loss, or anti-inflammatory cytokines (e.g., interleukin 10, interleukin 13 (IL-13), transforming growth factor-β) that promote bone formation and repair.^147,148^ Spaceflight-associated immune system weakening ultimately limits the ability of humans to expand their presence in space.^149^ Anemia and hematopoietic disorders are observed in astronauts, including leukocyte proliferation, a reduced number and activity of T-lymphocytes and natural killer cells, megakaryocyte loss and erythrocyte retention in the bone marrow compartment.^150,151^ Furthermore, the percentage of monocytes and macrophages has been shown to increase under simulated microgravity conditions.^152^ Interferon gamma (IFN-γ) is a potent proinflammatory activator of macrophages,^153^ and IL-4, IL-12, and IL-17 are key activators of inflammation.^154–156^ Notably, after spaceflight and ~24 h prior to landing, blood samples collected from 19 astronauts displayed significantly increased plasma levels of IL-4, IL-17, IL-1β, IL-12, TNFα and IFN-γ.^157–159^ The impact of microgravity on immune cell, blood lineage cell, and hematopoietic stem cell (HSC) dysfunction has not been extensively and systematically examined,^149^ and the responses in vivo are largely unknown. In terms of CD34^+^ HSCs, several studies have demonstrated their ability to enhance bone fracture repair.^160,161^ However, the role of HSCs and their combined effect with other elements of the hematopoietic niches in the bone healing process remain largely unknown.^162^ Microgravity has been shown to inhibit HSC proliferation,^163^ potentially due to slower cell cycle progression, in addition to inhibiting HSC migratory abilities, but with no loss in HSC self-renewal capacity.^164^ A microgravity-induced decrease in HSC differentiation to red blood cells has also been reported,^165^ and Shi et al.^149^ showed that microgravity significantly inhibited HSC differentiation to macrophages and impeded M1/2 polarization. The authors demonstrated that this effect involved the RAS/extracellular receptor kinase (ERK)/nuclear kappa-B ligand (NF-κβ) pathway and alterations in cellular metabolism. Hematopoietic stem cells are able to perceive both soluble signals and biomechanical inputs, including fluid mechanical stresses, from their microenvironment and are emerging as critical mechano-regulators of hematopoiesis.^151^ The beating of the heart subjects HSCs to constant hemodynamic forces. In mice, circulating HSCs can experience shear stress that exceeds 600 dyn per cm^2^ in regions of the aortic walls.^166^ However, adult HSCs sheltered in the bone marrow may not be exposed to blood flow directly. The HSC response to fluid shear may occur through the conversion of mechanical signals to protein-level expression via the mechanoresponsive transcription factor YAP. Recently, it was shown that YAP activation and the upregulation of YAP target genes are sensitive to cyclic stretch, and for the first time, a connection between biomechanical cues and YAP in determining HSC fate has been confirmed.^167^ Kruppel-like factor 2,^168^ basic leucine zipper,^151^ and cAMP response element-binding protein^169^ may all also serve as other crucial transcription factors whose expression reflects the onset of fluid shear forces and prompts HSC differentiation.

Indubitably, several cells other than MSCs, HSCs, osteoblasts, osteocytes, osteoclasts, and macrophages have critical responsibilities during healthy bone regeneration and repair (e.g., adipocytes, myocytes, and endothelial cells). These cells are also highly sensitive to microgravity and undergo morphological, functional, and biochemical changes in this environment.^150,170,171^ Adipocytes contribute to regulating bone formation via the promotion or inhibition of osteoblast and osteoclast differentiation through the expression and secretion of white adipose tissue-derived peptides (including leptin, adiponectin, vesfatin and resistin) and adipocytokines (such as TNFα, IL-6 and IL-1β).^172,173^ Notably, fluid flow has been shown to influence adipocyte maturation and activity. For example, when a physiological fluid shear stress of 10 dyn per cm^2^ at 1 Hz was applied for 1 h to 3T3-L1 murine preadipocytes, adipocyte maturation was suppressed.^174^ Interestingly, at a lower cyclical and continuous fluid shear stress of 0.77 dyn per cm^2^, human adipose stem cells (hASCs) displayed increased levels of osteogenic differentiation.^175^ Furthermore, hASCs exposed to much lower shear stresses (0.007 9, 0.031 3, and 0.078 6 dyn per cm^2^) and within 3D microfluidic devices showed decreased adiponectin secretion and increased free fatty acid secretion with increasing shear stress.^176^ This study showed that adipogenesis markers were downregulated as the shear stress increased, suggesting that extremely low fluid shear stresses may favor adipose tissue formation. Interestingly, Kim and colleagues^177^ applied a higher shear stress (19.8 dyn per cm^2^) to hASCs and reported the formation of endothelial cells. Together, these results suggest that fluid shear stress interactions with bone marrow adipocytes induce a mechanobiological response and that the responses can vary widely depending on the shear stresses applied.

Although not a focus in this review, the direct biochemical cross-talk between bone and skeletal muscle as a major driver of bone turnover is considered a novel research field.^178^ During unloading, many skeletal muscle factors (myokines) increase (e.g., IL-6, follistatin, olfactomedin1, and myostatin) and have detrimental effects on bone by supporting osteoclast formation and inhibiting osteoblast activity.^179,180^ For the first time, Takafuji et al.^181^ reported on the effects of fluid-driven mechanical stress on muscle-derived EV formation during muscle-bone interactions. Interestingly, the study demonstrated that application of a fluid flow shear stress of 6 dyn per cm^2^ to C2C12 cells significantly enhanced muscle cell-derived EV secretion that suppressed osteoclast formation and several osteoclast-related gene levels in both mouse bone marrow cells and macrophages.

Together, these studies demonstrate a lack of data; the further elucidation of the pathways involved in regulating bone turnover, including the role of adipocytes, macrophages, myocytes, and the HSC response to changes in fluid flow, is essential to more comprehensively understand healthy and pathological bone adaptation and regeneration in microgravity. The variable outcomes reported thus far indicate an intricate cellular response that demands careful systematic investigation to drive novel and critical therapeutic discoveries.

## The role of reactive oxygen species in the mechanoresponsive cell response to fluid flow in microgravity

Bone marrow mainly includes two types of cells with respect to their origin: hematopoietic and mesenchymal. Reactive oxygen species (ROS) critically regulate the fate and function of stem cells in both the nonhematopoietic (e.g., MSCs) and hematopoietic lineages (e.g., HSCs), thus tightly controlling bone hemostasis and turnover.^182^ Importantly, oxidative stress is implicated as a major causative factor in osteoporosis.^183^ Exposure to microgravity during spaceflight missions causes excessive ROS production that contributes to cellular stress and damage in astronauts.^184,185^ Oxidative stress causes protein and DNA damage,^186^ induces cell senescence,^187^ triggers osteoblast and osteocyte apoptosis and cell death,^188^ suppresses osteoblastogenesis, and promotes adipogenesis.^152,189^ Furthermore, osteoclasts are reported to be very sensitive to even low levels of ROS.^190–192^ Reactive oxygen species have been shown to decrease osteoprotegerin (OPG) expression and increase RANKL and TNFα secretion, osteoclastic differentiation,^193^ and ultimately bone resorption.^193,194^ This occurs through ERK and NF-κβ activation. Ultimately, these factors disrupt bone regeneration and repair^195,196^ and facilitate osteopenia and osteoporosis.^197^ Notably, many HLS rodent models used to simulate microgravity have reported increased intracellular oxidative stress, including within macrophages, MSCs and osteoblast-like cells.^198,199^ Furthermore, antioxidant treatment of HLS rats in vivo reduced intracellular ROS in macrophages. As a result, osteoclastic activity and bone density loss were significantly reduced, and bone structure and mechanical strength were preserved.^198–200^

The correlation between changes in fluid flow and ROS formation remains elusive. Several elements of the mechanotransduction cascade (e.g., ion channels, integrins, cytoskeletal network, receptor kinases, and membrane lipids^201^) are redox-sensitive, and the effect of ROS is likely disparate in each cell type. However, extremely few studies have investigated varying fluid flow and the subsequent ROS generation within bone cells, and this concept remains largely unexplored. Studies have investigated skeletal muscle cells and MSCs and demonstrated that varying the levels of applied stretch directly influenced whether a pro- or anti-ROS response was observed.^202–204^ Although not skeletally related, low fluid shear stress further increased endothelial intracellular ROS,^205,206^ downregulated ROS scavengers in endothelial cells,^207,208^ increased endothelial inflammatory (TNFα and IL-1β) cytokine release via NF-κβ,^206,209,210^ and caused apoptosis.^211^ In monocytes, flow-induced activation was shown to be regulated by ROS signaling,^212^ and upon activation by low fluid shear stress, these cells differentiated into inflammatory macrophages when investigated in a renal fluid shear model.^146^ Interestingly, Qin et al.^213^ recently demonstrated that low fluid shear stress facilitated the phagocytosis of EVs by vascular endothelial cells in vitro and in vivo and suggested that areas of low magnitude shear stress may provide a theoretical basis for the development of EV-based nanodrug delivery systems in vivo. Similarly, new approaches in clinical treatment are advancing, including the development of numerous novel mechanosensing carriers such as liposomes or microaggregates able to sense changes in shear force and respond by releasing biomolecules during mechanics-targeted drug delivery.^214,215^ Nevertheless, the studies described highlight the important need for further investigation into the role of fluid shear stress-induced ROS generation and its potentially direct relationship to the accelerated osteoporosis and dysfunction developed in microgravity. By exploiting fluid shear-mediated ROS pathways, there may be significant therapeutic potential for the treatment of diseases where oxidative stress plays a central role, such as osteoporosis.

## Fluid flow within osteoporotic bone and the potential role of adiposity in normogravity and microgravity

**Fig. 6:**
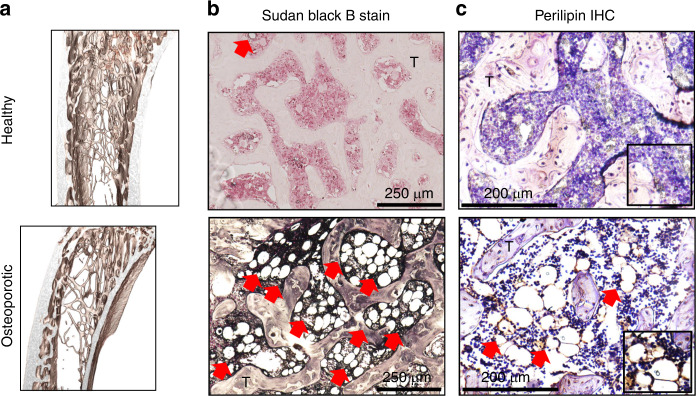
Images of healthy and osteoporotic bone in a rodent model developed in normogravity. A greater level of adiposity is observed within the osteoporotic architecture. **a** MicroCT images showing that the trabecular architecture is slowly resorbed, leading to alterations in pore number, size and shape. **b** Histological images demonstrate narrowing of the trabeculae (T) and the generation of an increased level of lipids in osteoporotic bone compared with healthy tissue. Sudan Black B stained phospholipids (gray) and intracellular lipids (black). **c** Perilipins are found exclusively on adipocytes, and using immunohistochemistry (IHC), a greater amount of positive staining is observed within the marrow of osteoporotic bone. Positive lipid staining is indicated by red arrows

Both osteoporosis on Earth and disuse-induced osteoporosis in microgravity progress by inducing a gradual transformation in bony macro- and microarchitecture where the interconnecting porous system is slowly resorbed. The porosity increases, and the pores enlarge with significant diversification in pore shape. These architectural alterations in pore number, size and shape, as well as an increased adiposity and viscosity within the bone marrow, not only affect the strength and stiffness of bone but also potentially modify fluid flow and the subsequent mechanical stimulus to cells (Fig. 6). Fat accumulation has not been verified in astronauts; however, bone marrow adiposity increased in rodents during spaceflight,^216,217^ and the causal relationship between increased adiposity and bone loss remains unclear. During spaceflight, functional hematopoietic tissue was replaced by marrow adipose tissue (MAT)^216^ in rats. Although MAT adipocytes may play a supporting role in CD34^+^ HSC proliferation^218^ and in the regeneration of MSCs and hematopoiesis,^219^ increasing MAT accumulation within bone is associated with a loss of HSCs, bone marrow dysfunction, high levels of ROS and proinflammatory cytokines, and the impairment of bone regeneration.^220,221^ Microgravity-induced brown adipose tissue may further contribute to the heightened metabolic dysfunction reported.^222,223^ Thus, MAT appears to have both osteoprotective and osteodestructive effects. Remarkably, Keune and colleagues^216^ reported that compared with ground controls, rats flown in space had a 32% lower cancellous bone area and 306% higher level of marrow adiposity. The increased adiposity was due to an increase in adipocyte number (224%) and size (26%). Interestingly, the authors reported no change in bone formation over the 14-day spaceflight. Zhang et al.^224^ demonstrated that under space microgravity, hMSCs favored adipogenesis over osteogenesis. These studies suggest that an overriding and dysfunctional response from MSCs, and potentially macrophages (via adipokine/proinflammatory cytokine release^225^) and osteoclasts, contributes to bone loss in microgravity. These alterations in bone architecture and marrow adiposity could limit new bone formation and favor bone resorption.^33,226^

**Fig. 7:**
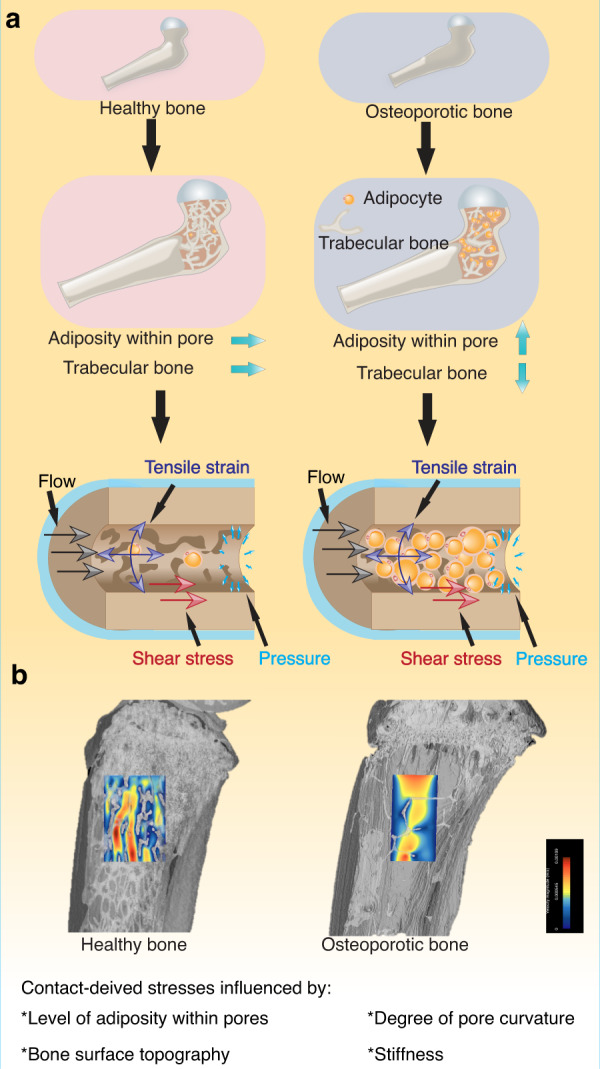
**a** A schematic demonstrating alterations in the stresses derived by fluid flow in healthy and osteoporotic bone. As fluid flows within the porous bone network, it imparts pressure, shear stress and tensile strain on cells within the local environment. These fluid-induced stresses are influenced by the degree of pore curvature, surface topography, stiffness and level of adiposity. The increased marrow adipocyte content that occurs in microgravity may lower the fluid-induced microstrain levels to the cells within the bone marrow, and this effect may be further amplified in microgravity as fluid flow is estimated to be reduced by up to 99.97%.^228^ It is conceivable that a reduced interstitial fluid flow combined with increased adiposity contributes to the accelerated bone loss observed in microgravity. **b** Using CFD modeling, disparate levels of fluid velocity were measured within the structure of healthy versus osteoporotic rat bone. Notably, the contribution of adiposity was not modeled. While these models identify changes in fluid flow within trabecular bone under these two conditions, more complex analyses are essential to determine the role of increasing adiposity and its role, if any, in shielding cells against fluid shear stress and accelerating bone loss

As osteoporosis progresses, the mechanical environment within bone becomes increasingly complex. It is theorized that in normogravity, deformation of the pores during loading induces motion in the fluid-like marrow, resulting in the generation of pressure and velocity gradients.^46^ Velocity gradients result in shear stress and tensile strain acting between the components of the marrow (Fig. 7). Using a computational fluid-structure interaction CFD model, Birmingham et al.^44^ demonstrated that in normogravity and under physiological loading conditions, the lower bone mass induced by osteoporosis resulted in an increase in fluid shear stresses to cells within the marrow. However, their results also estimated that due to the increased adipocyte formation that occurs as osteoporosis progresses, the viscosity of the bone marrow also decreases. This in turn reduced the shear stresses to cells, counteracting the increased architecturally induced stress measured. Furthermore, in one study, Metzger and colleagues^227^ used CFD and cyclic compression to estimate the shear stress to cells in the marrow. The results demonstrated that shear stress levels were amplified as the osteoporotic architecture deteriorated, with over 90% of nonadipocyte cells experiencing higher levels of shear stress. However, the maximum shear stress decreased by 20% when the more viscous osteoporotic marrow content was modeled. Similarly, Vaugh et al.^45^ developed a multiscale finite element model and demonstrated that a reduced bone volume resulted in an overall increase in bone deformation, leading to increased stimulation via microstrain to cells. Furthermore, an increased adipocyte content in the marrow resulted in lowering the microstrain levels to cells within the bone marrow, reportedly due to a shielding effect caused by the more compliant behavior of adipocytes. Despite this, the estimated levels of strain to cells remained much higher in the osteoporotic architecture; however, compensatory mechanobiological responses such as increased trabecular thickness and the axial alignment of trabeculae were effective in returning normal levels of microstrain to cells. In contrast, a poroelastic finite element analysis study by Gatti et al.^33^ showed reduced interstitial fluid flow within the LCS in osteoporotic rats when compared with healthy architecture. The influence of microgravity on fluid flow within bone has also been reported. Zhao et al.^228^ developed a 3D axisymmetric fluid-solid finite element model of bone with a two-stage pore structure. The results demonstrated a reduced fluid flow rate of up to 32.19% and that fluid shear stress decreased from ~2.0 × 10^−4^ Pa in normogravity to 1.6 × 10^−7^ to 6.0 × 10^−8^ Pa within the LCS in a microgravity field; this represents a decrease of 99.92% and 99.97%, respectively. The results also estimated that the flow velocity increased with gravitational acceleration. Nevertheless, these studies suggest that marrow viscosity and changes in the bone fraction volume, both within the larger pores and smaller LCS system, can directly affect mechanobiological signaling within bone. These studies also estimate that fluid transmission and the shear stresses to cells within the LCS are significantly dependent on the gravitational fields.

## In-orbit experimentation

Experiments in orbit are rare and extraordinarily costly, with many logistical challenges to overcome, and as a result, studies that investigate the biological effects of spaceflight are limited. Although several forms of ground-based devices have been designed to simulate weightlessness and the effect of microgravity on cells, simulated gravity still differs from the real state of microgravity during spaceflight. This is of particular relevance when considering the influence of fluid-imposed stresses and strains on cells. Under normogravity conditions, cells cultured in standard static conditions experience atmospheric pressure and hydrostatic pressure from the surrounding culture medium. Ground-based simulators such as the 1, 2 or 3D clinostat, rotating wall vessel, random positioning machine or use of diamagnetic levitation all directly or indirectly impact the fluid flow and/or stresses and strain imposed at the cellular level. Methods such as high-gradient magnetic fields (and subsequent force), vibration, centrifugal or rotational movement offer microgravity simulations that involve accelerated fluid motion where cells experience convection and flow-induced shear, friction, and other complex forces due to the movement of fluid molecules and cells against one another.^229,230^ Such forces will alter the cell-strain response, making the interpretation of results confounded and limited. The use of parabolic flight to achieve microgravity introduces interrupted moments of zero gravity, each lasting approximately 22 seconds. The control of fluid flow and subsequent changes in cell strain cannot be assessed within such short periods. Biological studies in parabolic flight are further complicated by the frequent change in gravity, which will expose both fluid and cells to a broad range of gravitational forces, making it impossible to decipher changes due to microgravity alone.

An additional and significant challenge facing scientists is the description and quantification of the migration forces and velocities at the single-cell level that occur during the rapid sedimentation of cells. For example, the density difference between red blood cells and plasma is approximately 0.1 g·cm^−3^, which leads to sedimentation velocities of several μm·s^−1^ for cells with a diameter of 7-8 µm.^231^ Therefore, subtle hydrodynamic effects under either gravity or microgravity are difficult to measure. At the cellular scale, tissue fluidity and mass transport depend on the dynamics of the cells in fluid flow, specifically on their deformation and orientation and the electrostatic forces that attract them together. These dynamics are governed by cellular rheological properties, such as internal viscosity and cytoskeleton elasticity. In diseases in which cell rheology is altered or the microenvironment is changed, tissue fluid flow may be severely impaired. The nonlinear interplay between cell rheology and flow may generate complex dynamics, which remain largely unexplored. Computational fluid dynamic models and technologies able to mimic cells and their flow and behavior are emerging. With a better understanding of 3D-based fluid mechanics at the micrometer-length scale, a new generation of experimental tools that provide control over cellular microenvironments able to emulate physiological conditions with exquisite accuracy offer an exciting and promising solution.

## Discussion

**Fig. 8:**
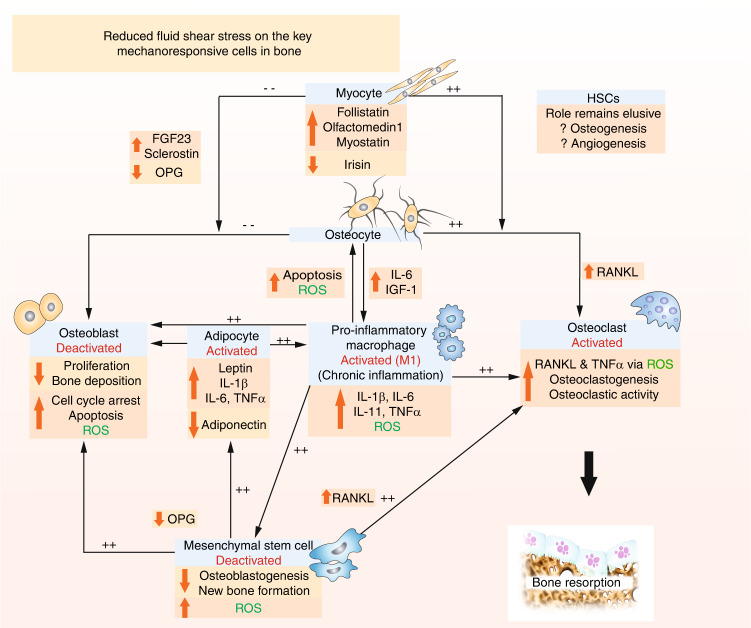
Based on the current literature, it is reasonable to speculate that extremely low fluid shear stresses induced via microgravity, alterations in osteoporotic bone architecture, and increased adiposity will together guide cells toward a phenotype that results in osteodestruction. *Hypothesized mechanisms* of bone loss due to extremely reduced fluid shear stress: osteocytes release more RANKL than OPG, thereby activating osteoclastic activity and a proinflammatory macrophage phenotype. The reduced OPG and increased proinflammatory cytokine release (e.g., IL-1β, IL-6, and TNFα) and ROS suppress the osteogenic differentiation of MSCs as well as the proliferation and deposition of new bone by osteoblasts. Increased myostatin and decreased irisin expression further activate osteoclastic activity and inhibit osteoblastic activity. MSCs preferentially differentiate into adipocytes (via increased leptin and decreased adiponectin, for example) while secreting proinflammatory cytokines that contribute to maintaining the M1 macrophage phenotype. It is conceivable that the concerted response of osteocytes, osteoclasts, macrophages, adipocytes, myocytes, and MSCs, among other key cells not shown (e.g., fibroblasts and endothelial cells), will serve to keep cells within a cycle of osteodestruction. The role of mechanoresponsive HSCs remains unknown. *Experimentally unconfirmed associations are labeled in green*

In recent years, our scientific interest in spaceflight has grown exponentially and resulted in a thriving area of research, with hundreds of astronauts spending months in space. Hypothetically, alterations in fluid shear stress, compression and tensile strain may alter mechanotransduction by reducing the mechano-stimulus to cells. Studies reveal that exposure to microgravity is estimated to result in a 99.97% decrease in shear stress to cells in the LCS and that this extreme condition causes loss of cilia and focal adhesions, stunted growth of microtubules and collapse of actin filaments. This dysfunction may be further amplified and expedited through increased adiposity developing within the bone marrow, shielding the cells from essential levels of mechanostimulation. Furthermore, reduced fluid flow with limited bulk convection may reduce larger solute transport, impact crystal and filament formation and cause unwanted biomolecule folding or aggregation, potentially leading to the creation of chronic long-term bone disease. It is conceivable that an independent and/or synergistic combination of these components may ultimately lead to reduced osteogenesis, increased osteoclastic resorption and the significant loss in bone structure, volume and strength witnessed in humans and animals when in microgravity (Fig. 8).

This review demonstrates that many questions remain. How and to what extent do changes in interstitial fluid flow and mass transport influence dysfunction? To what extent is reduced fluid velocity a contributing factor to bone loss within the osteoporotic microarchitecture? How soon does adiposity start to develop? It is known that it can take many years for bone mass to return when astronauts return to Earth, which suggests that the application of a “healthy” flow may not be able to restore dysfunctional cells to a normal phenotype. As commercial flights to space become accessible in the near future, many more humans will be exposed to space, conceivably with many in disparate states of health. A more sophisticated understanding of altered fluid flow and the role of gravity will undoubtedly accelerate new health safety strategies and solutions to the scientific challenges that remain, supporting both human exploration of space and human health on Earth.

## References

[1] Grigor’ev AI (2007). [Physiological problems of manned mission to Mars]. Ross. Fiziol. Zh . IM. IM. Sechenova.

[2] Grimm D (2015). The impact of microgravity on bone in humans. Bone.

[3] Moreno-Villanueva M, Wong M, Lu T, Zhang Y, Wu H (2017). Interplay of space radiation and microgravity in DNA damage and DNA damage response. npj Microgravity.

[4] Krause AR, Speacht TL, Zhang Y, Lang CH, Donahue HJ (2017). Simulated space radiation sensitizes bone but not muscle to the catabolic effects of mechanical unloading. PLoS One.

[5] Brügmann B (2018). Fundamentals of numerical relativity for gravitational wave sources. Science.

[6] Devahdhanush VS (2022). Experimental heat transfer results and flow visualization of vertical upflow boiling in Earth gravity with subcooled inlet conditions – In preparation for experiments onboard the International Space Station. Int. J. Heat. Mass Transf..

[7] Rezig M, Bellakhal G, Chahed J (2022). On turbulence and interfacial momentum transfer in dispersed gas-liquid flows: Contribution of bubbly flow experiments under microgravity conditions. Int. J. Multiph. Flow..

[8] Neely AN, Maley MP (2000). Survival of *Enterococci* and *Staphylococci* on hospital fabrics and plastic. J. Clin. Microbiol.

[9] Ashkarran AA, Suslick KS, Mahmoudi M (2020). Magnetically levitated plasma proteins. Anal. Chem..

[10] Norouzi N, Bhakta HC, Grover WH (2017). Sorting cells by their density. PLoS One.

[11] Pain RW (1977). Body fluid compartments. Anaesth. Intensive Care.

[12] Leeman M, Choi J, Hansson S, Storm MU, Nilsson L (2018). Proteins and antibodies in serum, plasma, and whole blood—size characterization using asymmetrical flow field-flow fractionation (AF4). Anal. Bioanal. Chem..

[13] Zhang Z, Witham S, Alexov E (2011). On the role of electrostatics in protein–protein interactions. Phys. Biol..

[14] Weber, C., Michaels, T., Mahadevan, L. Spatial control of irreversible protein aggregation. *Elife*. **8**, e42315 (2019). 10.7554/eLife.42315 PMC6516824 31084715

[15] Todd P (1989). Gravity-dependent phenomena at the scale of the single cell. ASGSB Bull..

[16] Coccarelli A, Boileau E, Parthimos D, Nithiarasu P (2016). An advanced computational bioheat transfer model for a human body with an embedded systemic circulation. Biomech. Model Mechanobiol..

[17] Ray L, Iliff JJ, Heys JJ (2019). Analysis of convective and diffusive transport in the brain interstitium. Fluids Barriers CNS.

[18] Kapellos, G. E. & Alexiou, T. S. Modeling momentum and mass transport in cellular biological media: from the molecular to the tissue scale. In: *Transport in Biological Media*. 1–40 (Elsevier, 2013).

[19] Swabb EA, Wei J, Gullino PM (1974). Diffusion and convection in normal and neoplastic tissues. Cancer Res..

[20] Waldeland JO, Evje S (2018). Competing tumor cell migration mechanisms caused by interstitial fluid flow. J. Biomech..

[21] Polacheck WJ, Charest JL, Kamm RD (2011). Interstitial flow influences direction of tumor cell migration through competing mechanisms. Proc. Natl. Acad. Sci..

[22] Yang, Y. & Leong, K. W. Microfluidic cell culture platforms with embedded nanoscale features. In: *Microfluidic Cell Culture Systems*. 3–26 (Elsevier, 2013).

[23] Chary SR, Jain RK (1989). Direct measurement of interstitial convection and diffusion of albumin in normal and neoplastic tissues by fluorescence photobleaching. Proc. Natl. Acad. Sci..

[24] Guevorkian, K., Brochard-Wyart, F., Gonzalez-Rodriguez, D. Flow dynamics of 3D multicellular systems into capillaries. In: *Viscoelasticity and Collective Cell Migration*. 193–223 (Elsevier, 2021).

[25] Wang L (2018). Solute transport in the bone lacunar-canalicular system (LCS). Curr. Osteoporos. Rep..

[26] Piekarski K, Munro M (1977). Transport mechanism operating between blood supply and osteocytes in long bones. Nature.

[27] Cummaudo M (2019). Histomorphometric analysis of osteocyte lacunae in human and pig: exploring its potential for species discrimination. Int. J. Leg. Med..

[28] Kameo Y, Adachi T, Sato N, Hojo M (2010). Estimation of bone permeability considering the morphology of lacuno-canalicular porosity. J. Mech. Behav. Biomed. Mater..

[29] Beno T, Yoon Y-J, Cowin SC, Fritton SP (2006). Estimation of bone permeability using accurate microstructural measurements. J. Biomech..

[30] Cowin SC, Cardoso L (2015). Blood and interstitial flow in the hierarchical pore space architecture of bone tissue. J. Biomech..

[31] Lovett M, Lee K, Edwards A, Kaplan DL (2009). Vascularization strategies for tissue engineering. Tissue Eng. Part B Rev..

[32] Weinbaum S, Cowin SC, Zeng Y (1994). A model for the excitation of osteocytes by mechanical loading-induced bone fluid shear stresses. J. Biomech..

[33] Gatti V, Azoulay EM, Fritton SP (2018). Microstructural changes associated with osteoporosis negatively affect loading-induced fluid flow around osteocytes in cortical bone. J. Biomech..

[34] Adachi T, Kameo Y, Hojo M (2010). Trabecular bone remodelling simulation considering osteocytic response to fluid-induced shear stress. Philos. Trans. R. Soc. A Math. Phys. Eng. Sci..

[35] Ganesh T, Laughrey LE, Niroobakhsh M, Lara-Castillo N (2020). Multiscale finite element modeling of mechanical strains and fluid flow in osteocyte lacunocanalicular system. Bone.

[36] Wu X (2016). Mathematically modeling fluid flow and fluid shear stress in the canaliculi of a loaded osteon. Biomed. Eng. Online.

[37] Liu H-Y (2020). Research on solute transport behaviors in the lacunar-canalicular system using numerical simulation in microgravity. Comput. Biol. Med..

[38] Wang H, Liu H, Wang X, Zhang C (2021). The lack of mass transfer in bone lacunar-canalicular system may be the decisive factor of osteoporosis under microgravity. Life Sci. Sp. Res..

[39] Verbruggen SW, Vaughan TJ, McNamara LM (2014). Fluid flow in the osteocyte mechanical environment: a fluid–structure interaction approach. Biomech. Model Mechanobiol..

[40] Price C, Zhou X, Li W, Wang L (2011). Real-time measurement of solute transport within the lacunar-canalicular system of mechanically loaded bone: Direct evidence for load-induced fluid flow. J. Bone Min. Res..

[41] Jansen LE, Birch NP, Schiffman JD, Crosby AJ, Peyton SR (2015). Mechanics of intact bone marrow. J. Mech. Behav. Biomed. Mater..

[42] Curtis KJ, Oberman AG, Niebur GL (2020). Effects of mechanobiological signaling in bone marrow on skeletal health. Ann. N. Y. Acad. Sci..

[43] Hu M (2013). Dynamic hydraulic fluid stimulation regulated intramedullary pressure. Bone.

[44] Birmingham E, Grogan JA, Niebur GL, McNamara LM, McHugh PE (2013). Computational modelling of the mechanics of trabecular bone and marrow using fluid structure interaction techniques. Ann. Biomed. Eng..

[45] Vaughan, T. J., Voisin, M., Niebur, G. L & McNamara, L. M. Multiscale modeling of trabecular bone marrow: understanding the micromechanical environment of mesenchymal stem cells during osteoporosis. *J. Biomech. Eng*. **37**, 10.1115/1.4028986 (2015). 10.1115/1.4028986 25363305

[46] Metzger TA, Schwaner SA, LaNeve AJ, Kreipke TC, Niebur GL (2015). Pressure and shear stress in trabecular bone marrow during whole bone loading. J. Biomech..

[47] Yao W, Li Y, Ding G (2012). Interstitial fluid flow: the mechanical environment of cells and foundation of meridians. Evid.-Based Complement Alter. Med..

[48] Mogilner A, Manhart A (2018). Intracellular fluid mechanics: coupling cytoplasmic flow with active cytoskeletal gel. Annu. Rev. Fluid Mech..

[49] Vogel V, Sheetz M (2006). Local force and geometry sensing regulate cell functions. Nat. Rev. Mol. Cell Biol..

[50] Klein-Nulend J, Bacabac R, Bakker A (2012). Mechanical loading and how it affects bone cells: the role of the osteocyte cytoskeleton in maintaining our skeleton. Eur. Cells Mater..

[51] Vogel V, Sheetz M (2006). Local force and geometry sensing regulate cell functions. Nat. Rev. Mol. Cell Biol..

[52] Alfieri R, Vassalli M, Viti F (2019). Flow-induced mechanotransduction in skeletal cells. Biophys. Rev..

[53] Herrmann M (2020). Interactions between muscle and bone—where physics meets biology. Biomolecules.

[54] Wang L (2020). Mechanical sensing protein PIEZO1 regulates bone homeostasis via osteoblast-osteoclast crosstalk. Nat. Commun..

[55] Sugimoto A (2017). Piezo type mechanosensitive ion channel component 1 functions as a regulator of the cell fate determination of mesenchymal stem cells. Sci. Rep..

[56] Tsimbouri PM (2017). Stimulation of 3D osteogenesis by mesenchymal stem cells using a nanovibrational bioreactor. Nat. Biomed. Eng..

[57] Li MCM, Chow SKH, Wong RMY, Qin L, Cheung WH (2021). The role of osteocytes-specific molecular mechanism in regulation of mechanotransduction – A systematic review. J. Orthop. Transl..

[58] Ohashi K, Fujiwara S, Mizuno K (2017). Roles of the cytoskeleton, cell adhesion and rho signalling in mechanosensing and mechanotransduction. J. Biochem..

[59] Wittkowske, C., Reilly, G. C., Lacroix, D., Perrault, C. M. In vitro bone cell models: impact of fluid shear stress on bone formation. *Front. Bioeng. Biotechnol*. **4**, 1–22 (2016). 10.3389/fbioe.2016.00087 PMC5108781 27896266

[60] Arnsdorf EJ, Tummala P, Kwon RY, Jacobs CR (2009). Mechanically induced osteogenic differentiation—the role of RhoA, ROCKII and cytoskeletal dynamics. J. Cell Sci..

[61] Pavalko FM (1998). Fluid shear-induced mechanical signaling in MC3T3-E1 osteoblasts requires cytoskeleton-integrin interactions. Am. J. Physiol..

[62] Malone AMD (2007). The role of actin cytoskeleton in oscillatory fluid flow-induced signaling in MC3T3-E1 osteoblasts. Am. J. Physiol. Physiol..

[63] Myers KA, Rattner JB, Shrive NG, Hart DA (2007). Osteoblast-like cells and fluid flow: cytoskeleton-dependent shear sensitivity. Biochem. Biophys. Res. Commun..

[64] Chen JC, Jacobs CR (2013). Mechanically induced osteogenic lineage commitment of stem cells. Stem Cell Res. Ther..

[65] Fritton SP, Weinbaum S (2009). Fluid and solute transport in bone: flow-induced mechanotransduction. Annu. Rev. Fluid Mech..

[66] Shi W (2020). Primary cilia act as microgravity sensors by depolymerizing microtubules to inhibit osteoblastic differentiation and mineralization. Bone.

[67] Jin J, Bakker AD, Wu G, Klein-Nulend J, Jaspers RT (2019). Physicochemical niche conditions and mechanosensing by osteocytes and myocytes. Curr. Osteoporos. Rep..

[68] Hinton PV, Rackard SM, Kennedy OD (2018). In Vivo Osteocyte Mechanotransduction: Recent Developments and Future Directions. Curr. Osteoporos. Rep..

[69] Haller, S. J. & Dudley, A. T. Extracellular mechanotransduction. *J. Gen. Physiol*. **154**, e202113026 (2022). 10.1085/jgp.202113026 PMC8855477 35171207

[70] Wang J, Lü D, Mao D, Long M (2014). Mechanomics: an emerging field between biology and biomechanics. Protein Cell.

[71] Jacobs CR (1998). Differential effect of steady versus oscillating flow on bone cells. J. Biomech..

[72] Liu L (2012). Different effects of intermittent and continuous fluid shear stresses on osteogenic differentiation of human mesenchymal stem cells. Biomech. Model Mechanobiol..

[73] Tan SD (2007). Osteocytes subjected to fluid flow inhibit osteoclast formation and bone resorption. Bone.

[74] Correia C, Bhumiratana S, Sousa RA, Reis RL, Vunjak-Novakovic G (2013). Sequential application of steady and pulsatile medium perfusion enhanced the formation of engineered bone. Tissue Eng. Part A.

[75] Bacabac RG (2004). Nitric oxide production by bone cells is fluid shear stress rate dependent. Biochem. Biophys. Res. Commun..

[76] Yi W (2010). Proteomic profiling of human bone marrow mesenchymal stem cells under shear stress. Mol. Cell Biochem..

[77] Kämmerer PW (2017). Cellular fluid shear stress on implant surfaces—establishment of a novel experimental set up. Int. J. Implant Dent..

[78] Anderson EJ, Falls TD, Sorkin AM, Tate MLK (2006). The imperative for controlled mechanical stresses in unraveling cellular mechanisms of mechanotransduction. Biomed. Eng. Online.

[79] Riehl BD, Lee JS, Ha L, Lim JY (2015). Fluid-flow-induced mesenchymal stem cell migration: role of focal adhesion kinase and RhoA kinase sensors. J. R. Soc. Interface.

[80] Grayson WL (2011). Optimizing the medium perfusion rate in bone tissue engineering bioreactors. Biotechnol. Bioeng..

[81] Johnson DL, McAllister TN, Frangos JA (1996). Fluid flow stimulates rapid and continuous release of nitric oxide in osteoblasts. Am. J. Physiol. Metab..

[82] Nauman EA, Satcher RL, Keaveny TM, Halloran BP, Bikle DD (2001). Osteoblasts respond to pulsatile fluid flow with short-term increases in PGE2 but no change in mineralization. J. Appl. Physiol..

[83] Yu W (2014). A microfluidic-based multi-shear device for investigating the effects of low fluid-induced stresses on osteoblasts. PLoS One.

[84] Leclerc E (2006). Study of osteoblastic cells in a microfluidic environment. Biomaterials.

[85] Jang K, Sato K, Igawa K, Chung U, Kitamori T (2008). Development of an osteoblast-based 3D continuous-perfusion microfluidic system for drug screening. Anal. Bioanal. Chem..

[86] Galbraith CG, Yamada KM, Sheetz MP (2002). The relationship between force and focal complex development. J. Cell Biol..

[87] Orr AW, Ginsberg MH, Shattil SJ, Deckmyn H, Schwartz MA (2006). Matrix-specific suppression of integrin activation in shear stress signaling. Mol. Biol. Cell.

[88] Jagodzinski M (2004). Effects of cyclic longitudinal mechanical strain and dexamethasone on osteogenic differentiation of human bone marrow stromal cells. Eur. Cells Mater..

[89] Qi M-C (2008). Mechanical strain induces osteogenic differentiation: Cbfa1 and Ets-1 expression in stretched rat mesenchymal stem cells. Int. J. Oral. Maxillofac. Surg..

[90] Huang CH, Chen MH, Young TH, Jeng JH, Chen YJ (2009). Interactive effects of mechanical stretching and extracellular matrix proteins on initiating osteogenic differentiation of human mesenchymal stem cells. J. Cell Biochem.

[91] Sumanasinghe RD, Bernacki SH, Loboa EG (2006). Osteogenic differentiation of human mesenchymal stem cells in collagen matrices: effect of uniaxial cyclic tensile strain on bone morphogenetic protein (BMP-2) mRNA expression. Tissue Eng..

[92] Yu H-S, Kim J-J, Kim H-W, Lewis MP, Wall I (2016). Impact of mechanical stretch on the cell behaviors of bone and surrounding tissues. J. Tissue Eng..

[93] Bhatt KA (2007). Uniaxial mechanical strain: an in vitro correlate to distraction osteogenesis. J. Surg. Res..

[94] Liegibel UM (2002). Concerted action of androgens and mechanical strain shifts bone metabolism from high turnover into an osteoanabolic mode. J. Exp. Med..

[95] Koike M, Shimokawa H, Kanno Z, Ohya K, Soma K (2005). Effects of mechanical strain on proliferation and differentiation of bone marrow stromal cell line ST2. J. Bone Min. Metab..

[96] Ward DF (2007). Mechanical strain enhances extracellular matrix-induced gene focusing and promotes osteogenic differentiation of human mesenchymal stem cells through an extracellular-related kinase-dependent pathway. Stem Cells Dev..

[97] Wang, Y. et al. Radiation induces primary osteocyte senescence phenotype and affects osteoclastogenesis in vitro. *Int. J. Mol. Med*. **47**, 76 (2021). 10.3892/ijmm.2021.4909 PMC7949628 33693957

[98] Dumas V (2009). The effect of dual frequency cyclic compression on matrix deposition by osteoblast-like cells grown in 3D scaffolds and on modulation of VEGF variant expression. Biomaterials.

[99] Jagodzinski M (2008). Influence of perfusion and cyclic compression on proliferation and differentiation of bone marrow stromal cells in 3-dimensional culture. J. Biomech..

[100] Liu J (2009). Hydrostatic pressures promote initial osteodifferentiation with ERK1/2 not p38 MAPK signaling involved. J. Cell Biochem..

[101] Anderson, E.J., Kaliyamoorthy, S., Knothe Tate, M.L. Modeling the effects of interstitial fluid flow on a single osteocyte and its processes. In: *Advances in Bioengineering*. 49–50 (ASMEDC, 2004).

[102] Sequeira Y, Maitra A, Pandey A, Jung S (2022). Revisiting the NASA surface tension driven convection experiments. npj Microgravity.

[103] Meseguer J (2014). Surface tension and microgravity. Eur. J. Phys..

[104] Jones SB, Or D (1999). Microgravity effects on water flow and distribution in unsaturated porous media: Analyses of flight experiments. Water Resour. Res..

[105] Yagi-Utsumi M (2020). Characterization of amyloid β fibril formation under microgravity conditions. npj Microgravity.

[106] DeLucas LJ (1986). Preliminary investigations of protein crystal growth using the space shuttle. J. Cryst. Growth.

[107] Martirosyan A (2019). Effect of macromolecular mass transport in microgravity protein crystallization. Gravitational Sp. Res..

[108] Bell D (2018). Self-assembly of protein fibrils in microgravity. Gravitational Sp. Res..

[109] Matsushita H (2021). Amyloid fibril formation is suppressed in microgravity. Biochem. Biophys. Rep..

[110] McPherson A, Malkin A, Kuznetsov Y (1995). The science of macromolecular crystallization. Structure.

[111] Tauber S (2017). Cytoskeletal stability and metabolic alterations in primary human macrophages in long-term microgravity. PLoS One.

[112] Tabony J, Job D (1992). Gravitational symmetry breaking in microtubular dissipative structures. Proc. Natl Acad. Sci..

[113] Tabony J, Job D (1990). Spatial structures in microtubular solutions requiring a sustained energy source. Nature.

[114] Mann V (2019). Changes in human foetal osteoblasts exposed to the random positioning machine and bone construct tissue engineering. Int J. Mol. Sci..

[115] Xu H (2017). Actin cytoskeleton mediates BMP2-Smad signaling via calponin 1 in preosteoblast under simulated microgravity. Biochimie.

[116] Buken C (2019). Morphological and molecular changes in juvenile normal human fibroblasts exposed to simulated microgravity. Sci. Rep..

[117] Thiel CS (2019). Real-time 3D high-resolution microscopy of human cells on the international space station. Int J. Mol. Sci..

[118] Nabavi N, Khandani A, Camirand A, Harrison RE (2011). Effects of microgravity on osteoclast bone resorption and osteoblast cytoskeletal organization and adhesion. Bone.

[119] Testa F (2014). Fractal analysis of shape changes in murine osteoblasts cultured under simulated microgravity. Rend. Lincei.

[120] Tabony J, Pochon N, Papaseit C (2001). Microtubule self-organisation depends upon gravity. Adv. Sp. Res..

[121] Hughes-Fulford M, Lewis ML (1996). Effects of microgravity on osteoblast growth activation. Exp. Cell Res..

[122] Tabony J (1994). Morphological bifurcations involving reaction-diffusion processes during microtubule formation. Science.

[123] Chen Z, Luo Q, Lin C, Kuang D, Song G (2016). Simulated microgravity inhibits osteogenic differentiation of mesenchymal stem cells via depolymerizing F-actin to impede TAZ nuclear translocation. Sci. Rep..

[124] Bradbury, P. et al. Modeling the impact of microgravity at the cellular level: implications for human disease. *Front. Cell Dev. Biol*. **8**, 10.3389/fcell.2020.00096 (2020). 10.3389/fcell.2020.00096 PMC7047162 32154251

[125] Di SM (2011). Graviresponses of osteocytes under altered gravity. Adv. Sp. Res..

[126] Louis F (2017). RhoGTPase stimulation is associated with strontium chloride treatment to counter simulated microgravity-induced changes in multipotent cell commitment. npj Microgravity.

[127] Corydon TJ (2016). Alterations of the cytoskeleton in human cells in space proved by life-cell imaging. Sci. Rep..

[128] Bauer J (2020). Microgravity and cell adherence. Int J. Mol. Sci..

[129] Hughes-Fulford M (2003). Function of the cytoskeleton in gravisensing during spaceflight. Adv. Sp. Res..

[130] Guignandon A (2001). Cell cycling determines integrin-mediated adhesion in osteoblastic ROS 17/2.8 cells exposed to space-related conditions. FASEB J..

[131] McCarthy ID (2005). Fluid shifts due to microgravity and their effects on bone: a review of current knowledge. Ann. Biomed. Eng..

[132] Baran R (2021). The cardiovascular system in space: focus on in vivo and in vitro studies. Biomedicines.

[133] Howden M, Siamwala JH, Hargens AR (2017). Bone microvascular flow differs from skin microvascular flow in response to head-down tilt. J. Appl. Physiol..

[134] Marshall-Goebel K (2019). Assessment of jugular venous blood flow stasis and thrombosis during spaceflight. JAMA Netw. Open.

[135] Hargens AR, Watenpaugh DE (1996). Cardiovascular adaptation to spaceflight. Med. Amp. Sci. Sport Amp. Exerc..

[136] Vernice NA, Meydan C, Afshinnekoo E, Mason CE (2020). Long-term spaceflight and the cardiovascular system. Precis Clin. Med..

[137] Colleran PN (2000). Alterations in skeletal perfusion with simulated microgravity: a possible mechanism for bone remodeling. J. Appl. Physiol..

[138] Klein-Nulend J, Bacabac RG, Veldhuijzen JP, Van Loon JJWA (2003). Microgravity and bone cell mechanosensitivity. Adv. Sp. Res..

[139] Amin S (2010). Mechanical factors and bone health: effects of weightlessness and neurologic injury. Curr. Rheumatol. Rep..

[140] Yang X, Sun L, Wang X, Fan Y (2010). Effects of simulated microgravity on the mechanosensibility of osteocytes due to fluid shear stress. Bone.

[141] Sun Z (2015). Simulated microgravity inhibits L-type calcium channel currents partially by the up-regulation of miR-103 in MC3T3-E1 osteoblasts. Sci. Rep..

[142] Gao X (2014). Regulation of cell migration and osteogenic differentiation in mesenchymal stem cells under extremely low fluidic shear stress. Biomicrofluidics.

[143] Port JR (2020). SARS-CoV-2 disease severity and transmission efficiency is increased for airborne but not fomite exposure in Syrian hamsters. Nat. Commun..

[144] Gao Y (2019). Migration and differentiation of osteoclast precursors under gradient fluid shear stress. Biomech. Model Mechanobiol..

[145] Seneviratne AN (2013). M1 macrophages are an early feature of shear stress modulated vulnerable atherosclerotic plaques. Eur. Heart J..

[146] Miravète M (2012). Renal tubular fluid shear stress facilitates monocyte activation toward inflammatory macrophages. Am. J. Physiol. Physiol..

[147] Yang D-H, Yang M-Y (2019). The role of macrophage in the pathogenesis of osteoporosis. Int J. Mol. Sci..

[148] Shapouri‐Moghaddam A (2018). Macrophage plasticity, polarization, and function in health and disease. J. Cell Physiol..

[149] Shi L (2021). Spaceflight and simulated microgravity suppresses macrophage development via altered RAS/ERK/NFκB and metabolic pathways. Cell Mol. Immunol..

[150] Blaber EA (2014). Mechanical unloading of bone in microgravity reduces mesenchymal and hematopoietic stem cell-mediated tissue regeneration. Stem Cell Res..

[151] Li H (2021). Biomechanical cues as master regulators of hematopoietic stem cell fate. Cell Mol. Life Sci..

[152] Dai S (2020). Effect of simulated microgravity conditions of hindlimb unloading on mice hematopoietic and mesenchymal stromal cells. Cell Biol. Int.

[153] Fajgenbaum DC, June CH (2020). Cytokine storm. N. Engl. J. Med..

[154] Junttila, I. S. Tuning the cytokine responses: an update on interleukin (IL)-4 and IL-13 receptor complexes. *Front. Immunol*. **9**, 888 (2018). 10.3389/fimmu.2018.00888 PMC6001902 29930549

[155] Milovanovic, J., et al. Interleukin-17 in chronic inflammatory neurological diseases. *Front. Immunol*. **11**, 947 (2020). 10.3389/fimmu.2020.00947 PMC7283538 32582147

[156] Balasubbramanian D, Goodlett BL, Mitchell BM (2019). Is IL-12 pro-inflammatory or anti-inflammatory? Depends on the blood pressure. Cardiovasc Res..

[157] Paulsen K (2015). Regulation of ICAM-1 in cells of the monocyte/macrophage system in microgravity. Biomed. Res. Int..

[158] Tauber S (2015). Signal transduction in primary human T lymphocytes in altered gravity during parabolic flight and clinostat experiments. Cell Physiol. Biochem..

[159] Crucian B (2013). Immune system dysregulation occurs during short duration spaceflight on board the space shuttle. J. Clin. Immunol..

[160] Marx RE, Harrell DB (2014). Translational research: The CD34^+^ cell is crucial for large-volume bone regeneration from the milieu of bone marrow progenitor cells in craniomandibular reconstruction. Int. J. Oral. Maxillofac. Implants.

[161] Kuroda R (2014). Clinical impact of circulating CD34-positive cells on bone regeneration and healing. Tissue Eng. Part B Rev..

[162] Oliveira CS, Carreira M, Correia CR, Mano JF (2022). The therapeutic potential of hematopoietic stem cells in bone regeneration. Tissue Eng. Part B Rev..

[163] Wang P (2019). Spaceflight/microgravity inhibits the proliferation of hematopoietic stem cells by decreasing Kit‐Ras/cAMP‐CREB pathway networks as evidenced by RNA‐Seq assays. FASEB J..

[164] Plett PA, Abonour R, Frankovitz SM, Orschell CM (2004). Impact of modeled microgravity on migration, differentiation, and cell cycle control of primitive human hematopoietic progenitor cells. Exp. Hematol..

[165] Zou L (2010). Simulated microgravity induce apoptosis and down-regulation of erythropoietin receptor of UT-7/EPO cells. Adv. Sp. Res..

[166] Suo J (2007). Hemodynamic shear stresses in mouse aortas. Arterioscler Thromb. Vasc. Biol..

[167] Lundin V (2020). YAP regulates hematopoietic stem cell formation in response to the biomechanical forces of blood flow. Dev. Cell.

[168] Wolfe RP, Ahsan T (2013). Shear stress during early embryonic stem cell differentiation promotes hematopoietic and endothelial phenotypes. Biotechnol. Bioeng..

[169] Ogawa H, Kozhemyakina E, Hung HH, Grodzinsky AJ, Lassar AB (2014). Mechanical motion promotes expression of Prg4 in articular cartilage via multiple CREB-dependent, fluid flow shear stress-induced signaling pathways. Genes Dev..

[170] Morbidelli L (2005). Simulated hypogravity impairs the angiogenic response of endothelium by up-regulating apoptotic signals. Biochem. Biophys. Res. Commun..

[171] Cialdai F (2017). Modeled microgravity affects fibroblast functions related to wound healing. Microgravity Sci. Technol..

[172] Muruganandan S, Govindarajan R, Sinal CJ (2018). Bone marrow adipose tissue and skeletal health. Curr. Osteoporos. Rep..

[173] Shin E, Koo JS (2020). The role of adipokines and bone marrow adipocytes in breast cancer bone metastasis. Int. J. Mol. Sci..

[174] Choi J, Lee SY, Yoo Y M, Kim CH (2017). Maturation of adipocytes is suppressed by fluid shear stress. Cell Biochem. Biophys..

[175] Elashry MI, Gegnaw ST, Klymiuk MC, Wenisch S, Arnhold S (2019). Influence of mechanical fluid shear stress on the osteogenic differentiation protocols for Equine adipose tissue-derived mesenchymal stem cells. Acta Histochem.

[176] Yang F (2021). A 3D human adipose tissue model within a microfluidic device. Lab Chip.

[177] Kim HW, Lim J, Rhie JW, Kim DS (2017). Investigation of effective shear stress on endothelial differentiation of human adipose-derived stem cells with microfluidic screening device. Microelectron. Eng..

[178] Lau P, Vico L, Rittweger J (2022). Dissociation of bone resorption and formation in spaceflight and simulated microgravity: potential role of myokines and osteokines. Biomedicines.

[179] Juhl OJ (2021). Update on the effects of microgravity on the musculoskeletal system. npj Microgravity.

[180] Kawao N, Morita H, Iemura S, Ishida M, Kaji H (2020). Roles of Dkk2 in the linkage from muscle to bone during mechanical unloading in mice. Int. J. Mol. Sci..

[181] Takafuji Y (2021). Effects of fluid flow shear stress to mouse muscle cells on the bone actions of muscle cell-derived extracellular vesicless. PLoS One.

[182] Li X, Li B, Shi Y, Wang C, Ye L (2021). Targeting reactive oxygen species in stem cells for bone therapy. Drug Disco. Today.

[183] Kimball JS, Johnson JP, Carlson DA (2021). Oxidative stress and osteoporosis. J. Bone Jt Surg..

[184] Gómez X (2021). Key points for the development of antioxidant cocktails to prevent cellular stress and damage caused by reactive oxygen species (ROS) during manned space missions. npj Microgravity.

[185] Ran F, An L, Fan Y, Hang H, Wang S (2016). Simulated microgravity potentiates generation of reactive oxygen species in cells. Biophys. Rep..

[186] Li N, An L, Hang H (2015). Increased sensitivity of DNA damage response-deficient cells to stimulated microgravity-induced DNA lesions. PLoS One.

[187] Davalli P, Mitic T, Caporali A, Lauriola A, D’Arca D (2016). ROS, Cell senescence, and novel molecular mechanisms in aging and age-related diseases. Oxid. Med. Cell Longev..

[188] Ray PD, Huang B-W, Tsuji Y (2012). Reactive oxygen species (ROS) homeostasis and redox regulation in cellular signaling. Cell Signal.

[189] Atashi F, Modarressi A, Pepper MS (2015). The role of reactive oxygen species in mesenchymal stem cell adipogenic and osteogenic differentiation: a review. Stem Cells Dev..

[190] Koh JM (2006). Homocysteine enhances bone resorption by stimulation of osteoclast formation and activity through increased intracellular ROS generation. J. Bone Min. Res..

[191] Moon HJ (2011). Simvastatin inhibits osteoclast differentiation by scavenging reactive oxygen species. Exp. Mol. Med..

[192] Harrison C (2013). Targeting NOX4 knocks down osteoporosis. Nat. Rev. Drug Discov..

[193] Agidigbi TS, Kim C (2019). Reactive oxygen species in osteoclast differentiation and possible pharmaceutical targets of ROS-mediated osteoclast diseases. Int. J. Mol. Sci..

[194] Tao H (2020). ROS signaling cascades: dual regulations for osteoclast and osteoblast. Acta Biochim. Biophys. Sin..

[195] Domazetovic V (2017). Oxidative stress in bone remodeling: role of antioxidants. Clin. Cases Min. Bone Metab..

[196] Almeida M, O’Brien CA (2013). Basic biology of skeletal aging: role of stress response pathways. J. Gerontol. Ser. A Biol. Sci. Med. Sci..

[197] Angeloni C, Maraldi T, Vauzour D (2014). Redox signaling in degenerative diseases: from molecular mechanisms to health implications. Biomed. Res. Int..

[198] Xin M (2015). Attenuation of hind-limb suspension-induced bone loss by curcumin is associated with reduced oxidative stress and increased vitamin D receptor expression. Osteoporos. Int..

[199] Morikawa D (2013). Cytoplasmic reactive oxygen species and SOD1 regulate bone mass during mechanical unloading. J. Bone Min. Res..

[200] Colaianni G (2017). Irisin prevents and restores bone loss and muscle atrophy in hind-limb suspended mice. Sci. Rep..

[201] Hsieh H-J, Liu C-A, Huang B, Tseng AH, Wang DL (2014). Shear-induced endothelial mechanotransduction: the interplay between reactive oxygen species (ROS) and nitric oxide (NO) and the pathophysiological implications. J. Biomed. Sci..

[202] Pardo PS, Mohamed JS, Lopez MA, Boriek AM (2011). Induction of Sirt1 by mechanical stretch of skeletal muscle through the early response factor EGR1 triggers an antioxidative response. J. Biol. Chem..

[203] Chen X (2018). Mechanical stretch induces antioxidant responses and osteogenic differentiation in human mesenchymal stem cells through activation of the AMPK-SIRT1 signaling pathway. Free Radic. Biol. Med..

[204] Dick AS (2013). Cyclic stretch stimulates nitric oxide synthase-1-dependent peroxynitrite formation by neonatal rat pulmonary artery smooth muscle. Free Radic. Biol. Med..

[205] Chao Y (2018). Low shear stress induces endothelial reactive oxygen species via the AT1R/eNOS/NO pathway. J. Cell Physiol..

[206] Li B, Zhang J, Wang Z, Chen S (2016). Ivabradine prevents low shear stress induced endothelial inflammation and oxidative stress via mTOR/eNOS pathway. PLoS One.

[207] Brooks AR, Lelkes PI, Rubanyi GM (2002). Gene expression profiling of human aortic endothelial cells exposed to disturbed flow and steady laminar flow. Physiol. Genomics.

[208] Mueller CFH (2005). The role of the multidrug resistance protein-1 in modulation of endothelial cell oxidative stress. Circ. Res..

[209] Chao Y (2017). Inhibition of angiotension II type 1 receptor reduced human endothelial inflammation induced by low shear stress. Exp. Cell Res..

[210] Ishibazawa A, Nagaoka T, Yokota H, Ono S, Yoshida A (2013). Low shear stress up-regulation of proinflammatory gene expression in human retinal microvascular endothelial cells. Exp. Eye Res..

[211] Zhang J (2013). Low shear stress induces human vascular endothelial cell apoptosis by activating Akt signal and increasing reactive oxygen species. Nan Fang. Yi Ke Da Xue Xue Bao.

[212] Sorescu GP (2004). Bone morphogenic protein 4 produced in endothelial cells by oscillatory shear stress induces monocyte adhesion by stimulating reactive oxygen species production from a Nox1-based NADPH oxidase. Circ. Res..

[213] Qin X (2022). Uptake of oxidative stress-mediated extracellular vesicles by vascular endothelial cells under low magnitude shear stress. Bioact. Mater..

[214] Wang J, Kaplan JA, Colson YL, Grinstaff MW (2017). Mechanoresponsive materials for drug delivery: harnessing forces for controlled release. Adv. Drug Deliv. Rev..

[215] Wang J, Colson YL, Grinstaff MW (2018). Tension-activated delivery of small molecules and proteins from superhydrophobic composites. Adv. Health. Mater..

[216] Keune JA, Philbrick KA, Branscum AJ, Iwaniec UT, Turner RT (2016). Spaceflight-induced vertebral bone loss in ovariectomized rats is associated with increased bone marrow adiposity and no change in bone formation. npj Microgravity.

[217] Endicott, J., Fitzgerald, J. Increased bone marrow adiposity in murine femoro-tibial epiphyses exposed to 30 days of microgravity. *Matters Sel*. 10.19185/matters.201904000010 (2019).

[218] Poloni A (2013). Molecular and functional characterization of human bone marrow adipocytes. Exp. Hematol..

[219] Zhou BO (2017). Bone marrow adipocytes promote the regeneration of stem cells and haematopoiesis by secreting SCF. Nat. Cell Biol..

[220] Ambrosi TH (2017). Adipocyte accumulation in the bone marrow during obesity and aging impairs stem cell-based hematopoietic and bone regeneration. Cell Stem Cell.

[221] Miggitsch C (2019). Human bone marrow adipocytes display distinct immune regulatory properties. EBioMedicine.

[222] Chen Y (2019). Simulated microgravity led to increased brown adipose tissue activity in rats. Acta Astronaut.

[223] Wong CP, Iwaniec UT, Turner RT (2021). Evidence for increased thermogenesis in female C57BL/6J mice housed aboard the international space station. npj Microgravity.

[224] Zhang C (2018). Space microgravity drives transdifferentiation of human bone marrow‐derived mesenchymal stem cells from osteogenesis to adipogenesis. FASEB J..

[225] Ouchi N, Parker JL, Lugus JJ, Walsh K (2011). Adipokines in inflammation and metabolic disease. Nat. Rev. Immunol..

[226] Ciani C, Sharma D, Doty SB, Fritton SP (2014). Ovariectomy enhances mechanical load-induced solute transport around osteocytes in rat cancellous bone. Bone.

[227] Metzger TA, Vaughan TJ, McNamara LM, Niebur GL (2017). Altered architecture and cell populations affect bone marrow mechanobiology in the osteoporotic human femur. Biomech. Model Mechanobiol..

[228] Zhao S (2020). Numerical analysis of the flow field in the lacunar-canalicular system under different magnitudes of gravity. Med. Biol. Eng. Comput..

[229] Consolo F (2012). Computational modeling for the optimization of a cardiogenic 3D bioprocess of encapsulated embryonic stem cells. Biomech. Model Mechanobiol..

[230] Marsano A (2006). Use of hydrodynamic forces to engineer cartilaginous tissues resembling the non-uniform structure and function of meniscus. Biomaterials.

[231] Podgorski, T., Coupier, G., Minetti, C. Red blood cell dynamics: the contribution of microgravity in the BIOMICS project. In: *Preparation of Space Experiments*. (IntechOpen, 2020). 10.5772/intechopen.93471.

[232] Xing J (2014). Surface chemistry modulates osteoblasts sensitivity to low fluid shear stress. J. Biomed. Mater. Res Part A.

[233] Li J, Rose E, Frances D, Sun Y, You L (2012). Effect of oscillating fluid flow stimulation on osteocyte mRNA expression. J. Biomech..

[234] Haasper C (2008). Cyclic strain induces FosB and initiates osteogenic differentiation of mesenchymal cells. Exp. Toxicol. Pathol..

[235] Shi Y (2011). Continuous cyclic mechanical tension inhibited Runx2 expression in mesenchymal stem cells through RhoA-ERK1/2 pathway. J. Cell Physiol..

[236] Fang, B. Osteogenic response of mesenchymal stem cells to continuous mechanical strain is dependent on ERK1/2-Runx2 signaling. *Int. J. Mol. Med*. **29**, 1083–1089 (2012). 10.3892/ijmm.2012.934 22407386

[237] Wu T (2021). Involvement of mechanosensitive ion channels in the effects of mechanical stretch induces osteogenic differentiation in mouse bone marrow mesenchymal stem cells. J. Cell Physiol..

[238] Stavenschi E, Corrigan MA, Johnson GP, Riffault M, Hoey DA (2018). Physiological cyclic hydrostatic pressure induces osteogenic lineage commitment of human bone marrow stem cells: a systematic study. Stem Cell Res. Ther..

[239] Ravichandran A (2017). In vitro cyclic compressive loads potentiate early osteogenic events in engineered bone tissue. J. Biomed. Mater. Res Part B Appl Biomater..

[240] Chen X (2017). Cyclic compression stimulates osteoblast differentiation via activation of the Wnt/β-catenin signaling pathway. Mol. Med. Rep..

[241] Wang D, Wang H, Gao F, Wang K, Dong F (2017). ClC‐3 promotes osteogenic differentiation in MC3T3‐E1 cell after dynamic compression. J. Cell Biochem..

[242] Nagatomi J, Arulanandam BP, Metzger DW, Meunier A, Bizios R (2003). Cyclic pressure affects osteoblast functions pertinent to osteogenesis. Ann. Biomed. Eng..

[243] Priam S (2013). Identification of soluble 14-3-3∊ as a novel subchondral bone mediator involved in cartilage degradation in osteoarthritis. Arthritis Rheum..

[244] Liu C (2010). Effects of cyclic hydraulic pressure on osteocytes. Bone.

[245] Sittichockechaiwut A, Scutt AM, Ryan AJ, Bonewald LF, Reilly GC (2009). Use of rapidly mineralising osteoblasts and short periods of mechanical loading to accelerate matrix maturation in 3D scaffolds. Bone.

[246] Kikuta J, Yamaguchi M, Shimizu M, Yoshino T, Kasai K (2015). Notch signaling induces root resorption via RANKL and IL-6 from hPDL cells. J. Dent. Res..

[247] Mosley JR, Lanyon LE (1998). Strain rate as a controlling influence on adaptive modeling in response to dynamic loading of the ulna in growing male rats. Bone.

[248] Rubin CT, Sommerfeldt DW, Judex S, Qin Y-X (2001). Inhibition of osteopenia by low magnitude, high-frequency mechanical stimuli. Drug Discov. Today.

[249] Nagaraja M, Jo H (2014). The role of mechanical stimulation in recovery of bone loss—high versus low magnitude and frequency of force. Life.

[250] Frost HM (1987). Bone mass and the mechanostat: A proposal. Anat. Rec..

